# Neuroblastoma Tyrosine Kinase Signaling Networks Involve FYN and LYN in Endosomes and Lipid Rafts

**DOI:** 10.1371/journal.pcbi.1004130

**Published:** 2015-04-17

**Authors:** Juan Palacios-Moreno, Lauren Foltz, Ailan Guo, Matthew P. Stokes, Emily D. Kuehn, Lynn George, Michael Comb, Mark L. Grimes

**Affiliations:** 1 Division of Biological Sciences, Center for Structural and Functional Neuroscience, University of Montana, Missoula, Montana, United States of America; 2 Cell Signaling Technology, Inc., Danvers, Massachusetts, United States of America; 3 Department of Neuroscience, Johns Hopkins University School of Medicine, Baltimore, Maryland, United States of America; 4 Department of Cell Biology and Neuroscience, Montana State University, Bozeman, Montana, United States of America; Pierre and Marie Curie University (UPMC), France

## Abstract

Protein phosphorylation plays a central role in creating a highly dynamic network of interacting proteins that reads and responds to signals from growth factors in the cellular microenvironment. Cells of the neural crest employ multiple signaling mechanisms to control migration and differentiation during development. It is known that defects in these mechanisms cause neuroblastoma, but how multiple signaling pathways interact to govern cell behavior is unknown. In a phosphoproteomic study of neuroblastoma cell lines and cell fractions, including endosomes and detergent-resistant membranes, 1622 phosphorylated proteins were detected, including more than half of the receptor tyrosine kinases in the human genome. Data were analyzed using a combination of graph theory and pattern recognition techniques that resolve data structure into networks that incorporate statistical relationships and protein-protein interaction data. Clusters of proteins in these networks are indicative of functional signaling pathways. The analysis indicates that receptor tyrosine kinases are functionally compartmentalized into distinct collaborative groups distinguished by activation and intracellular localization of SRC-family kinases, especially FYN and LYN. Changes in intracellular localization of activated FYN and LYN were observed in response to stimulation of the receptor tyrosine kinases, ALK and KIT. The results suggest a mechanism to distinguish signaling responses to activation of different receptors, or combinations of receptors, that govern the behavior of the neural crest, which gives rise to neuroblastoma.

## Author Summary

Neuroblastoma is a childhood cancer for which therapeutic progress has been slow. We analyzed a large number phosphorylated proteins in neuroblastoma cells to discern patterns that indicate functional signal transduction pathways. To analyze the data, we developed novel techniques that resolve data structure and visualize that structure as networks that represent both protein interactions and statistical relationships. We also fractionated neuroblastoma cells to examine the location of signaling proteins in different membrane fractions and organelles. The analysis revealed that signaling pathways are functionally and physically compartmentalized into distinct collaborative groups distinguished by phosphorylation patterns and intracellular localization. We found that two related proteins (FYN and LYN) act like central hubs in the tyrosine kinase signaling network that change intracellular localization and activity in response to activation of different receptors.

## Introduction

Neuroblastoma arises from cells of the neural crest, a population of multipotent, migrating cells that differentiate into neurons in the peripheral nervous system, melanocytes, and structural cells [1]. Neuroblastoma represents 7–10% of childhood cancers and about half of all infant cancers. Positive prognosis ranges from 95% to 10% depending on age, markers expressed in tumor cells, and stage of progression. 70% of neuroblastomas are already metastatic at diagnosis. There is compelling evidence that stalled or incomplete cell differentiation is the primary defect that gives rise to this cancer [2–6]. Neural crest cells appear to restrict their range of cell fate choices in sequential steps [7,8], and the profound heterogeneity in neuroblastoma is caused by a failure to differentiate at different stages. Neuroblastoma tumors and cell lines thus represent a snapshot of failed differentiation at different stages in the neural crest sympathoadrenal lineage [2,4,7,8]. Anaplastic lymphoma kinase (ALK), a receptor tyrosine kinase (RTK), is frequently mutated and activated in both familial and spontaneous neuroblastomas, suggesting that this receptor can prevent a key differentiation step in neural crest cells [9–15]. Incompletely differentiated cells may give rise to a proliferating population when mutations occur that allow checkpoints in the cell division cycle and mechanisms of programmed cell death to be bypassed. The tragic outcome is too often a metastatic cancer with poor prognosis.

To address this clinically challenging problem, a greater understanding of the signaling mechanisms that are active in neural crest and neuroblastoma is required. Tyrosine kinase signaling networks play a major role in governing cell differentiation, including in neuroblastoma [16]. There are 90 tyrosine kinases in the human genome; 58 of these are receptor tyrosine kinases [17,18], many of which have unknown functions. Src Homology 2 (SH2) domains (and one-fifth of phosphotyrosine-binding or PTB domains) mediate selective protein–protein interactions with proteins phosphorylated on tyrosine residues, and thus mediate assembly of phosphotyrosine signaling networks [19]. The metazoan evolution of multicellular organisms coincided with expansion of tyrosine kinases, protein tyrosine phosphatases, and SH2 domains, which suggests that tyrosine kinase signaling mechanisms play a major role in cell differentiation [20–22]. Unfortunately, the system isn’t foolproof, and cancer results when the dynamic assembly of signaling complexes goes awry [23]. Thus, the complexity of kinase-substrate and other protein-protein interactions in tyrosine kinase signaling pathways is important to understand because these pathways govern the choice between differentiation and cancer.

Tyrosine kinase signaling mechanisms are intimately intertwined with mechanisms that govern protein interactions in endocytosis. Src Homology 3 (SH3) domains are among the most abundant protein domain modules encoded by eukaryotic genomes; over 300 SH3 domains are found in 213 human proteins [24,25]. SH3 domain-containing proteins, which typically bind to proline-rich motifs [26], are functionally linked to both endocytosis and tyrosine kinase signaling pathways [24]. SH3-domain-containing proteins play a role in endocytosis that is conserved in yeast, worms, and humans [26,27]. SH3 proteins may also contain other domains (e.g., kinase, phosphatase, GTP exchange, GTPase activating) to perform conserved functions in endocytosis and cytoskeletal dynamics, and, in metazoans, RTK signaling [28,29]. 36 human proteins contain one SH2 domain and one or more SH3 domain(s) (SH2-SH3 proteins) [25]. Most SH2-SH3 proteins are phosphorylated on multiple sites on tyrosine as well as serine and/or threonine residues. Half of them also have tyrosine kinase domains, e.g., the SRC-family kinases (SFKs). Interactions between proteins that contain SH2 and SH3 domains indicate that tyrosine kinase signaling and endocytosis are linked, and there is good evidence that endocytosis and signal transduction in general are integrated [30,31].

To identify patterns in tyrosine phosphorylation in neuroblastoma, we acquired phosphoproteomic data from 21 neuroblastoma cell lines and cell fractions including endosomes and detergent-resistant lipid rafts as previously characterized [32,33]. New approaches were devised to analyze these data. We previously experimented with different dimensionality reduction and clustering techniques and validated methods that effectively resolve clusters from lung cancer phosphoproteomic data [34]. An important first step is to represent missing values as “data not available” instead of zero in spectrometry data. By combining pattern recognition techniques with gene ontology (GO) and protein-protein interaction (PPI) data, we learned that clusters that contain interacting proteins are likely to indicate functional signaling pathways [34–40]. Here, we extend methods that employ graph theory and pattern recognition algorithms to introduce techniques to visualize data structure, namely a cluster-filtered network (CFN) and co-cluster correlation network (CCCN). We focussed primarily on proteins containing tyrosine kinase, tyrosine phosphatase, SH2 and SH3 domains, which collectively we call phosphotyrosine network control proteins (PNCPs).

## Results

### Phosphoproteomics

To identify patterns in tyrosine phosphorylation in neuroblastoma, we analyzed tyrosine phosphoproteomic data acquired from 21 neuroblastoma cell lines using immunoprecipitation of tyrosine phosphorylated peptides as previously described [41,42]. Four cell lines [SH-SY5Y, LAN-6, SMS-KCN, and SK-N-BE(2)] were selected for further studies because of their different point mutations in ALK, p53 status, RTK expression, morphology, and growth patterns. These cells were fractionated to isolate endosomes and detergent-resistant lipid rafts [32,33], and analyzed under different conditions that changed the state of their signaling pathways. Quantification of immunoprecipitated phosphopeptides was obtained from the peak intensity of each peptide (from the MS1 spectrum of the intact peptide before fragmentation for MS/MS analysis) [41,42].

We experimented with different ways to analyze the mass spectrometry data (described in detail in Materials and Methods). For the first analysis described below, phosphopeptide amounts were summed for each protein in each sample, with the exception of the SRC-family kinases (SFKs), where the C-terminal inhibitory phosphorylation was summed separately and given the names SRC_i; LYN_i; FYN_i; and YES1_i. This provided an overview of which proteins were present and phosphorylated together in the same samples. For the second analysis, phosphopeptides were summed into individual phosphorylation sites, which were then clustered. Clustering data were obtained by treating all samples mathematically as different states in the neuroblastoma system. We describe analysis of the whole dataset first, then subsets of the data, focusing on signaling proteins in endosomes and detergent-resistant membranes.

### Neuroblastoma Phosphoproteomic Network

1622 phosphorylated proteins were identified in all neuroblastoma samples (S1 Fig; S3 Dataset). 1203 of these were tyrosine phosphorylated, identified from peptides immunoprecipitated using an anti-phosphotyrosine antibody. 557 proteins were identified from phospho-AKT substrate immunoprecipitation; of these 419 were unique, and 138 were dually phosphorylated proteins also found in the phosphotyrosine data. Due to limits in mass spectrometric detection of peptides [43–47], these data were not an exhaustive determination of all phosphorylated proteins in all samples. To ask whether these data were complete enough for analysis of signaling pathways, we employed graph theory, which describes the properties of networks [35,38]. S1 Fig shows a network constructed using proteins identified in neuroblastoma phosphoproteomic data as nodes, and protein-protein interaction (PPI) edges merged as described [34]. We found that the entire neuroblastoma phosphoproteomic network of 1622 proteins and 18728 interactions is dense enough to have the structure and properties expected of biological networks, including clusters that can be usefully interpreted (S2 Fig). PPI databases are biased towards proteins best studied in the scientific literature [36–38], and not all protein-protein interactions in PPI databases may occur in neuroblastoma cells. Nevertheless, PPI network analysis indicates that the phosphoproteomic data are complete enough to examine further to gain insight into signal transduction pathways that are active in neuroblastoma (S2 Fig).

We hypothesize that proteins containing tyrosine kinase, tyrosine phosphatase, SH2 and SH3 domains (PNCPs) will collectively initiate and control phosphotyrosine signaling pathways [19,24]. In neuroblastoma phosphoproteomic data, we detected 31 phosphorylated RTKs out of 58 in the human genome (S3 Fig); 41 of 110 SH2-domain-containing proteins; 12 out of 38 (or 107 possible, based on open reading frames in the human genome) proteins containing the tyrosine phosphatase (PTPc) domain; and 61 out of the 216 human SH3-domain containing proteins. There are 36 proteins in the human genome that contain both SH2 and SH3 domains and 17 of these were detected in neuroblastoma phosphoproteomic data.

These data indicate that neuroblastoma cell lines express and phosphorylate a large fraction of the PNCPs in the human genome. This remarkable diversity in phosphotyrosine signaling pathways likely represents a snapshot of signaling pathways activated in the sympathoadrenal lineage of neural crest that gives rise to neuroblastoma at different stages of development [2–6]. The robust expression of RTK pathways that are known to function in neural crest differentiation suggests the hypothesis that neuroblastoma cells might be multipotent despite being selected for proliferation in culture. To test this hypothesis we transplanted neuroblastoma cells in to the developing neural tube of live chick embryos and indeed found that they were capable of both migration and terminal differentiation (S4 Fig). Notably, four different transplanted human neuroblastoma cell lines [LAN6, SK-N-BE(2), SMS-KCN, and SH-SY5Y] migrated to neural crest target sites, incorporated into the developing ganglia, and expressed neuronal markers specific to mature afferents (S4 Fig). The potential to migrate along the stereotypical neural crest migration pathways, and differentiate into most neural-crest-derived cell types, suggests that many of the RTK signaling pathways that control differentiation and migration were generally functional in these neuroblastoma cell lines. Thus, our phosphoproteomic data has relevance to pathways active in neural crest from which neuroblastoma is derived, and warrants detailed analysis.

### Embedding and Cluster Analysis

We developed new methods to analyze proteomic data based on the hypothesis that data structure can be described using a combination of graph theory and pattern recognition techniques. The first key step was to recognize that missing data, which are common in mass spectrometry data due to stochastic variation in phosphopeptide detection, should not have a value of zero [34]. The next key step was to represent different statistical relationships by proximity on two- or three-dimensional graphs using an effective dimension reduction, or embedding, technique, t-distributed stochastic neighbor embedding (t-SNE) [48,49]. Clusters were identified by proximity on resulting three-dimensional data structures (embeddings) using a minimum spanning tree, single linkage method [34,50]. 75–80 clusters were identified from each embedding based on dissimilarity calculated in different ways (*S1 Movie; S1 Dataset*). Clusters were evaluated internally, based on the primary data, and externally, using PPI and gene ontology (GO) databases (S5 Fig). These evaluations confirm that these methods effectively resolve meaningful clusters as previously described [34].

We experimented with different approaches to use these clusters to define signaling pathways active in neuroblastoma. One approach was to apply a “hard” filter, or exclusive approach to identify groups of proteins that co-cluster from two or more dissimilarity representations. This exclusive approach separates groups of proteins that are most likely to define core units of signaling pathways [34]. Alternatively, an inclusive approach treats clusters derived from different embeddings as equally valid and therefore allows overlap between cluster membership. This inclusive approach recognizes that signaling pathways use common effectors. We show results from each of these approaches in turn.

**Fig 1:**
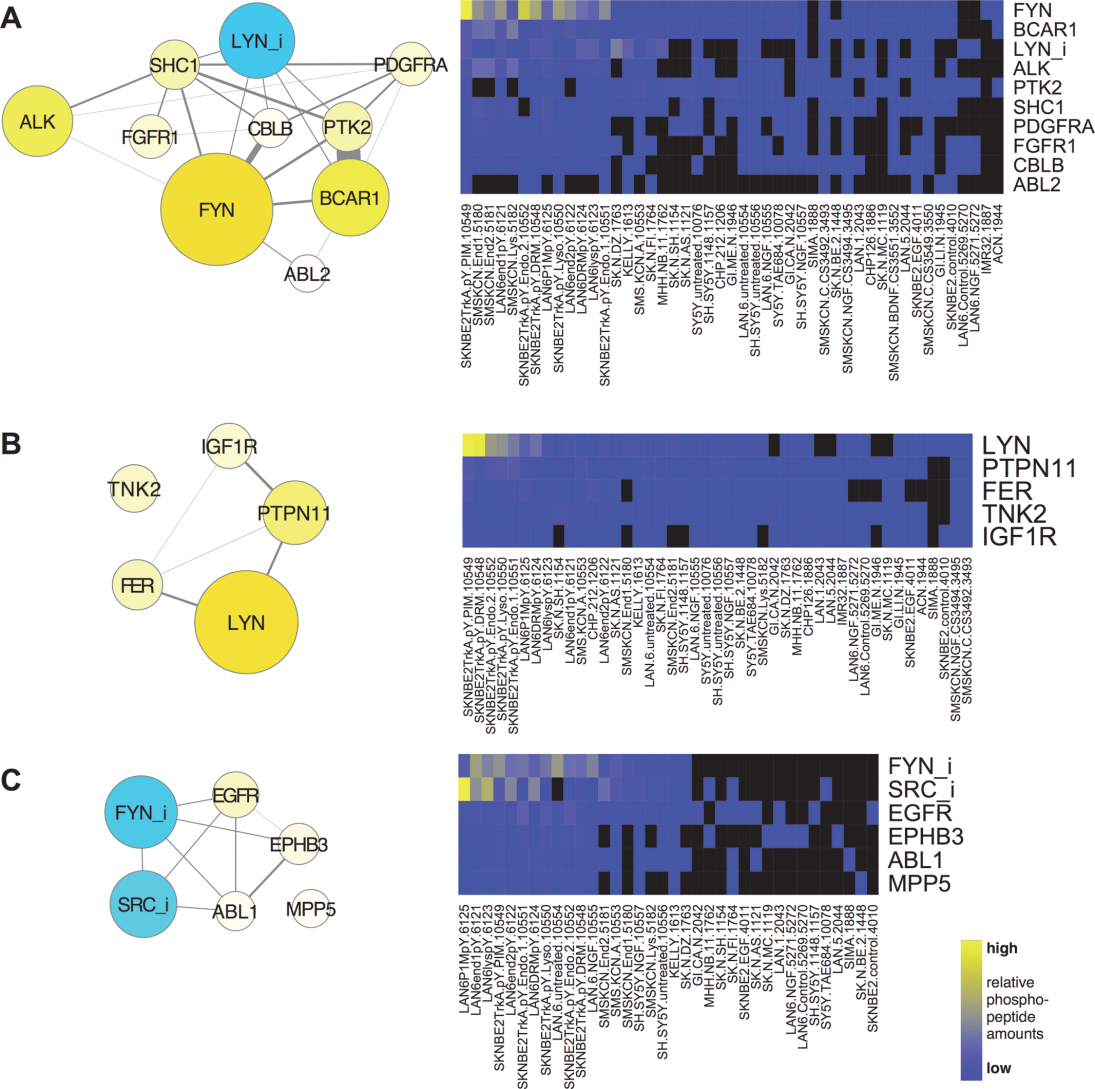
Proteins that cluster with RTKs. Proteins with tyrosine kinase, tyrosine phosphatase, SH2, and/or SH3 domains (PNCPs) that co-clustered with ALK (**A**), IGF1R (**B**), and EGFR (**C**) by t-SNE using both Spearman *and* Euclidean dissimilarity representations (“hard” filter), graphed as networks with PPI edges (left) and heat maps (right). The size and color of nodes is scaled to graph total phosphopeptides detected for each protein; blue represents phosphorylation on inhibitory sites (*e*.*g*., SFKs labelled “SFK_i”), yellow, all other sites. Heat maps (right) display the relative total phosphopeptide amounts for each protein on a blue-yellow scale (black represents NA; key, bottom right), sorted most to least left to right and top to bottom for samples and proteins, respectively, in each cluster.

For the first, exclusive cluster analysis, we focused on PNCPs and proteins whose phosphorylation pattern was statistically most similar determined by both Euclidean distance and Spearman correlation (Figs 1 and S6). Heat maps (Fig 1 and S6, right) indicate that the phosphorylation patterns in the primary data are reasonably consistent within each cluster. The RTK, ALK, clustered with two other RTKs (FGFR1, PDGFRA), activated FYN, and LYN phosphorylated on the C-terminal inhibitory site (LYN_i; Fig 1A). The tyrosine kinase, FAK (PTK2), and the adaptor molecules BCAR1, SHC1 and CBLB were included in this group of PNCPs. We also noted other clusters that suggest interactions among phosphorylated tyrosine kinases: IGF1R with LYN, FER, the phosphatase PTPN11/SHP-2, and the tyrosine kinase TNK2, whose interactions with other proteins in this group have not been previously characterized (Fig 1B). In addition, we found that EGFR and EPHB3 clustered with inhibited FYN and SRC as well as the SH3, SH2 containing tyrosine kinase, ABL1, and MPP5, a protein with PDZ, SH3, and guanylate kinase domains whose interactions are not characterized (Fig 1C). Examples of other clusters identified using this hard filter are shown in S6 Fig. These clusters define phosphorylated proteins most commonly phosphorylated together in the same samples in this data set, which suggests possible interactions among signaling proteins that were previously unknown. Assignment of proteins to one cluster should not be viewed as evidence for excluding it from participating in a signaling pathway identified in another cluster, however [34].

An alternative, inclusive approach is to recognize possible relationships defined by different measures of statistical similarity. Clusters derived from t-SNE applied to Spearman, Euclidean, and hybrid Spearman-Euclidean (SED) embeddings were typically overlapping but not identical, yet reasonably close in their ability to resolve meaningful clusters as determined by external and internal evaluations (S5 Fig; [34]). This suggests that statistical relationships independently defined by Euclidean distance or Spearman correlation are equally valid. Using this inclusive method that recognizes clusters derived from different embeddings had the advantage that it allows overlap between cluster membership, which makes sense biologically for these data because signaling pathways overlap and converge.

**Fig 2:**
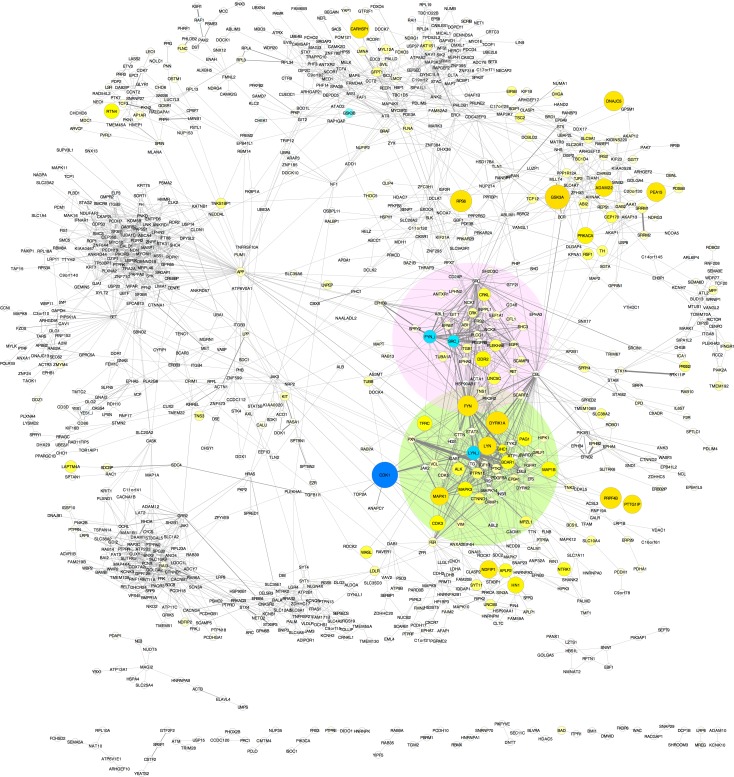
The neuroblastoma cluster-filtered network (CFN). Edges from the neuroblastoma network shown in S1 Fig were filtered to show only edges among proteins that co-clustered based on Spearman, Euclidean, *or* hybrid Spearman-Euclidean dissimilarity. Nodes are graphed as in Fig 1 using an edge-weighted, spring-embedded layout. Small isolated groups with limited interactions are at the bottom of the figure. Proteins that have no interaction edges within clusters are not shown. PNCPs in aggregated, co-clustering collaborative groups, indicated by shaded regions, are displayed in Fig 3. (The dark blue node next to the region shaded in green is CDK1 phosphorylated on its inhibitory site.)

We employed the inclusive clustering strategy to filter protein interaction edges to obtain the cluster-filtered network (CFN) shown in Fig 2. In this graph, only edges among proteins that co-clustered based on Spearman, Euclidean, or hybrid Spearman-Euclidean (SED) dissimilarity are shown. This CFN data structure is useful because graph layouts that treat edges like springs (edge-weighted, spring embedded; force-directed) aggregate proteins that share a statistical relationship *and* interact with one another, so nearest neighbors are likely to represent functional groups (regions highlighted in Fig 2).

An alternative visualization of data structure is a co-cluster correlation network (CCCN; S7 Fig). In this graph, edges represent positive (yellow) or negative (blue) correlation, filtered to show only edges among proteins that clustered together and have a Spearman correlation coefficient greater than the absolute value of 0.5. The networks in Figs 2 and S7 are complementary because they apply a different filter to clustering results. Proteins that interact with one another may not tightly correlate, and co-clustered proteins that do tightly correlate may not have been studied previously for evidence of interactions. These filtered networks thus prune cluster members that have no evidence for interaction and do not tightly correlate with others in the group, yet allow potential interactions among pathways to be studied because overlapping cluster membership is defined by different embeddings.

**Fig 3:**
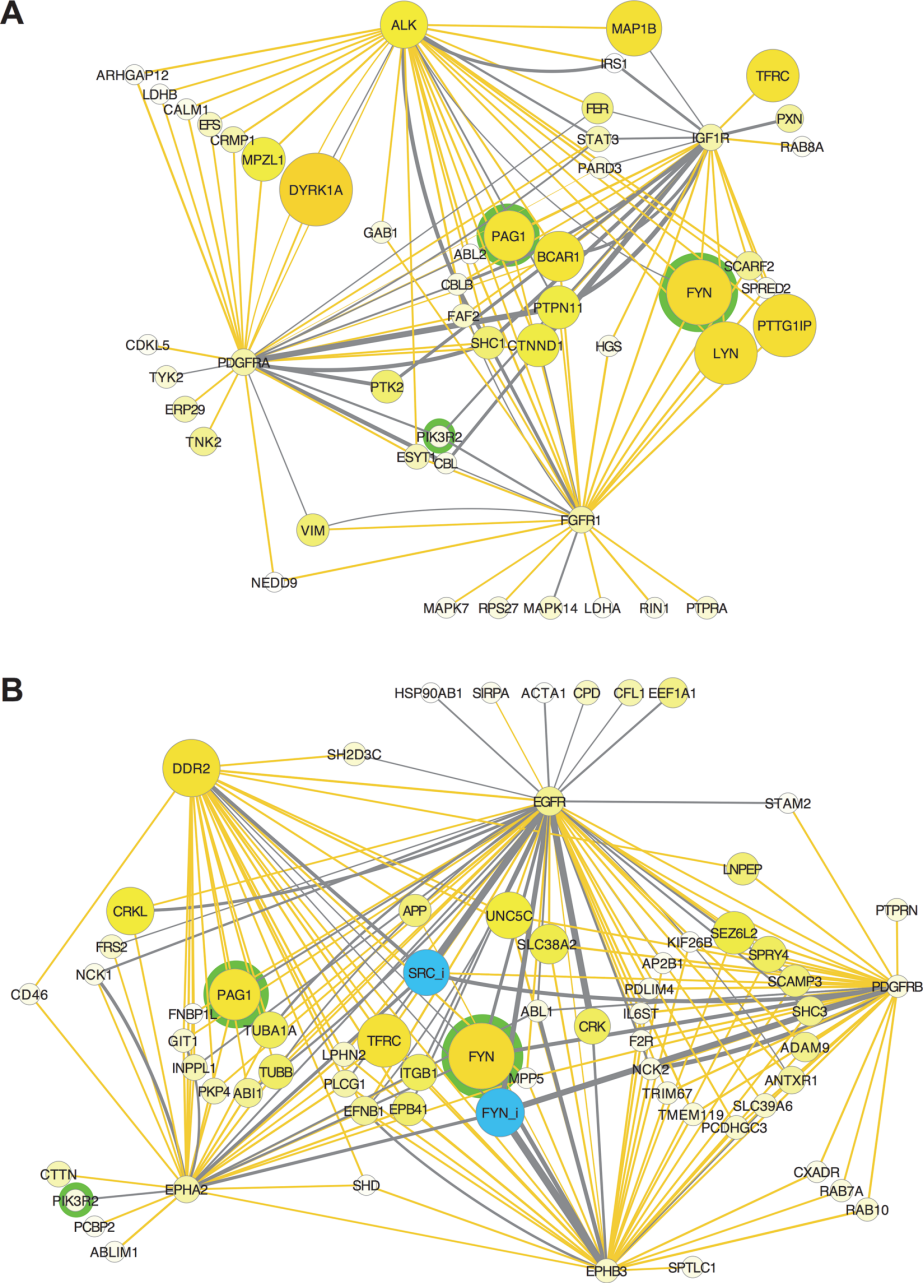
Collaborative groups of RTKs. Interaction networks show proteins that co-cluster and have Spearman correlation > 0.5 (yellow edges) or are known to interact from PPI databases (grey edges) with ALK, PDGFRA, FGFR1, and IGFR1 **(A)**; EGFR, DDR2, EPHA2, EPHB3, and PDGFRB **(B)**. Only edges linked to RTKs are shown. Proteins are grouped by the number of interactions with RTKs (*e*.*g*. the group in the center containing PAG1 and BCAR1 in **A** interacts with all four RTKs). Nodes were filtered to exclude those with lowest representation in the phosphoproteomic data. Node size and color indicates total phosphorylation as in Fig 2. Proteins that are both known to interact and have a positive correlation have two edges (*e*.*g*. ALK edges connecting FYN, SHC1, and IRS1). PAG1, FYN, and PIK3R2, the only nodes in common to both networks, are highlighted in green.

Exploration of these networks reveals potential functional interactions among signaling proteins defined by the structure of neuroblastoma phosphoproteomic data. We noted two groups of highly phosphorylated RTKs that clustered together (Fig 3). Networks in Fig 3 show only positive correlation (yellow) and PPI (grey) edges between RTKs and co-clustered effector proteins, with proteins that link to three or more receptors grouped in the center of the graphs (Fig 3). The similarity in phosphorylation patterns for proteins in these groups can be seen in heat maps of the primary data (S8 Fig). Co-clustering of ALK with PDGFRA, FGFR1, and IGF1R (through co-clustering with FGFR1) is indicative of a collaborative relationship (Fig 3A). Similarly, EGFR co-clusters with PDGFRB, EPHA2, EPHB3, and DDR2 (Fig 3B), indicating that these RTKs form a separate collaborative group. While different RTKs *within* these collaborative groups share a number of co-clustering downstream proteins in common, the only effector proteins in common *between* these two collaborative groups are PIK3R2, FYN, and the SFK scaffold protein, PAG1 [51].

The following general conclusions can be made from these analyses so far. Clusters that contain proteins that interact with one another, identified using statistical relationships from phosphoproteomic data, likely indicate functional signaling pathways. New potential interactions are suggested when strong clustering is observed among proteins whose physical interactions have not been previously characterized (*e*.*g*., TNK2 and MPP5 in Fig 1). Common patterns of phosphorylation in neuroblastoma samples suggests collaboration among RTKs within functional groups (Fig 3). Since activation of different RTKs was associated with different states of activation and inhibition of different SFKs, particularly FYN and LYN (Figs 1 and 3), we next examined how stimulation or inhibition of RTKs affected phosphorylation of other tyrosine kinases.

### Tyrosine Kinase Posphorylation in Response to RTK Stimulation

**Fig 4:**
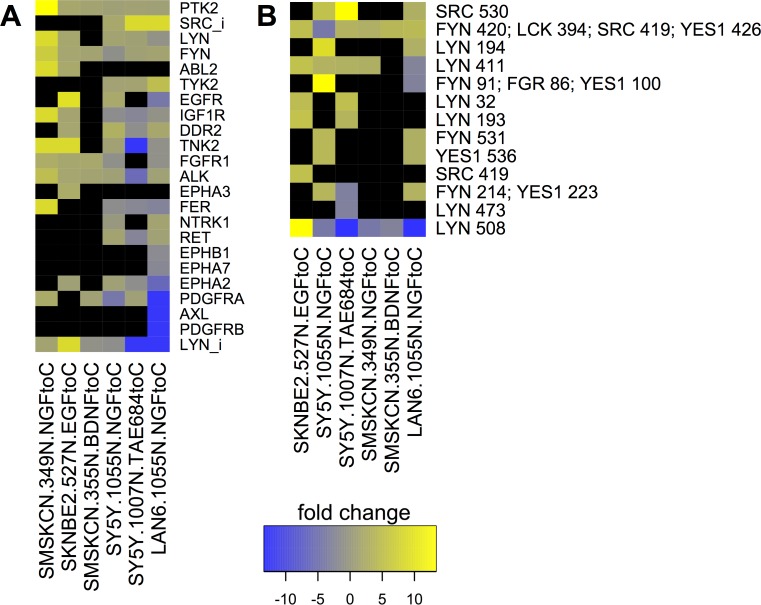
Fold change in response to RTK stimulation or inhibition. Shown are changes of more than twofold from representative experiments where peak intensity was measured for treatment and control conditions in the same experiment with cell lines and treatments indicated on column labels (*e*.*g*., “NGF to C” means NGF-treated compared to control). (**A**) Total phosphorylation changes in tyrosine kinases. SFKs phosphorylated on their C-terminal inhibitory site were tracked separately (SFK_i). In addition to results summarized in the text, the ALK inhibitor, TAE684, inhibited RET and IGF1R phosphorylation about threefold in SH-SY5Y cells. NGF stimulated phosphorylation of IGF1R and PDGFRA, and BDNF treatment increased phosphorylation of FGFR1, in SMS-KCN cells. EGFR and EPHA2 were affected in opposite ways in LAN-6 and SH-SY5Y cells. AXL, PDGFRB, EPHA7, and EPHB1 phosphorylation were decreased by NGF in LAN6 cells. Individual phosphorylation site changes are shown for SFKs (**B**). Activating (SFK Y411-426) and inhibitory (SFK Y508-531) sites on FYN, LYN, YES1, and SRC were affected differently by different treatments. Phosphorylation sites represent the sum of all peptides surrounding that site; peptides whose conserved sequence is present in several proteins are indicated with multiple names, *e*.*g*., “FYN 420; LCK 394; SRC 419; YES1 426.” Fold changes are graphed on a blue-yellow color scale with blue representing a decrease, and yellow, an increase, compared to control (key). Data are sorted from most to least for each total row (protein or phosphorylation site) and column (treatment) from left to right and top to bottom, respectively.

RTK activation affects other RTKs, SFKs, and other tyrosine kinases. To examine the effects of RTK stimulation on other tyrosine kinases, we compared phosphoproteomic data from cells treated to influence RTK activity, or not treated, in the same experiment. Fig 4A shows tyrosine kinases whose total phosphorylation changed more than two-fold under experimental conditions where RTKs were stimulated by ligand or ALK was inhibited. For example, NGF treatment caused a more than twofold increase in total phosphorylation of DDR2, and more than fivefold decrease in phosphorylation of PDGFRA in both LAN-6 and SH-SY5Y cells. EGF treatment of SK-N-BE(2) cells activated EGFR and stimulated EPHA3 phosphorylation about 3-fold (Fig 4A). These data indicate that stimulation of one RTK affects the phosphorylation state of other RTKs in neuroblastoma cell lines.

Changes in inhibitory phosphorylation of LYN and SRC were also observed (Fig 4A, LYN_i; SRC_i), so individual phosphorylation sites on SFKs and other kinases were examined further. Phosphopeptides were assigned to phosphorylation sites based on peptide sequence homology (see Materials and Methods). The data revealed that both activating (SFK Y411-426) and inhibitory (SFK Y508-531) phosphorylation sites on the SFKs LYN, FYN, YES1, and SRC were significantly affected in different ways by treatments that influence RTK activity (Fig 4B). For example, the LYN inhibitory phosphorylation (LYN 508) was reduced by NGF treatment and increased by EGF treatment. In contrast, FYN inhibitory phosphorylation (FYN 531) was increased by NGF in two cell lines (Fig 4B). These data suggest the hypothesis that activation and inhibition of LYN and FYN distinguishes responses to different RTKs (Figs 1 and 3).

Tyrosine phosphorylation of RTKs is generally thought to be a measure of activation, but differences in different RTK phosphorylation sites were seen in these experiments. For example, NGF treatment both increased and decreased phosphorylation on different sites on EGFR, RET, IGF1R, ALK, and other RTKs in LAN-6 and SH-SY5Y cells (S9A Fig). Some variations in individual phosphorylation site responses to treatments were also observed for other tyrosine kinases (S9B Fig), but they were not as dramatic as those of SFKs (Fig 4B).

These data indicate that different RTKs initiate signaling mechanisms to cause distinct phosphorylation patterns on other tyrosine kinases, including RTKs and SFKs. Combined with the clustering patterns shown in Figs 1 and 3, the data suggest the hypothesis that SFKs, particularly FYN and LYN, discern and integrate signals from different RTKs. We hypothesized that functional interactions among these signaling proteins may occur in specific intracellular locations, namely endosomes and lipid rafts, and therefore we performed phosphoproteomic analyses on these fractions.

### Endosomes and Detergent Resistant Membranes

We asked whether particular signaling proteins were enriched in endosomes and detergent-resistant membranes (DRMs). RTKs are present in endosomes that can be distinguished from other types of receptors by size and density (S10 Fig) [32]. Phosphoproteomic analysis was also performed on detergent-resistant and-sensitive fractions distinguished by extraction with non-ionic detergent (S10 Fig) [33,52].

**Fig 5:**
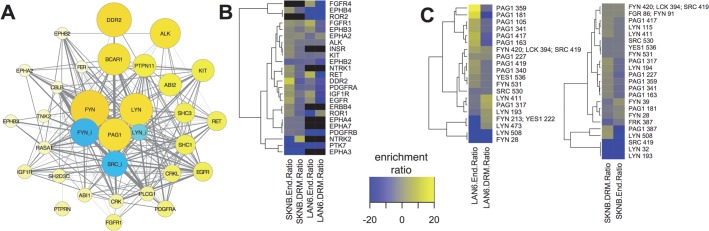
PNCP enrichment in endosomes and DRM fractions. (**A**) The most highly phosphorylated PNCPs that were present in endosome fractions from two or more cell lines, graphed as in Fig 1 except node size and color intensity represents total phosphorylation in endosome fractions. (**B**) Enrichment of proteins in endosome and DRM fractions was calculated as the ratio of amounts in endosomes or DRMs vs. the average in all other fractions and samples from that cell line, graphed as a heat map. (**C**) SFK and PAG1 phosphorylation site enrichment in LAN-6 cells (left) and SK-N-BE(2) cells (right).

Endosomes from three neuroblastoma cell lines were characterized by phosphoproteomic analysis. In all endosome fractions from three cell lines (LAN-6, SMS-KCN, SK-N-BE(2)), 908 proteins were detected, including 22 RTKs, 10 tyrosine phosphatases; 30 SH2- and 44 SH3-domain-containing proteins. The most highly phosphorylated RTKs in neuroblastoma were those identified in Fig 5A by large yellow nodes that indicates large amounts detected in endosome fractions (*e*.*g*., DDR2, ALK, KIT, RET, EGFR, PDGFA, FGFR1). FYN and LYN containing both activating and inhibiting phosphorylations were also prominent in endosomes, along with PAG1, inhibited SRC (SRC_i), the SH3 adaptor protein BCAR1, several other adaptor proteins, two tyrosine phosphatases (PTPN11/SHP-2 and PTPRN), and PLCG1/PLCγ1, which was found previously in endosomes in PC12 cells [53]. Notably, 26 out of the 55 SH3-domain-containing proteins in the human genome that were predicted to have a function in endocytosis based on orthologous interactions in *C*. *elegans* were found in neuroblastoma endosome fractions, and 2 of the 55 were detected in lysosome fractions [24].

**Fig 6:**
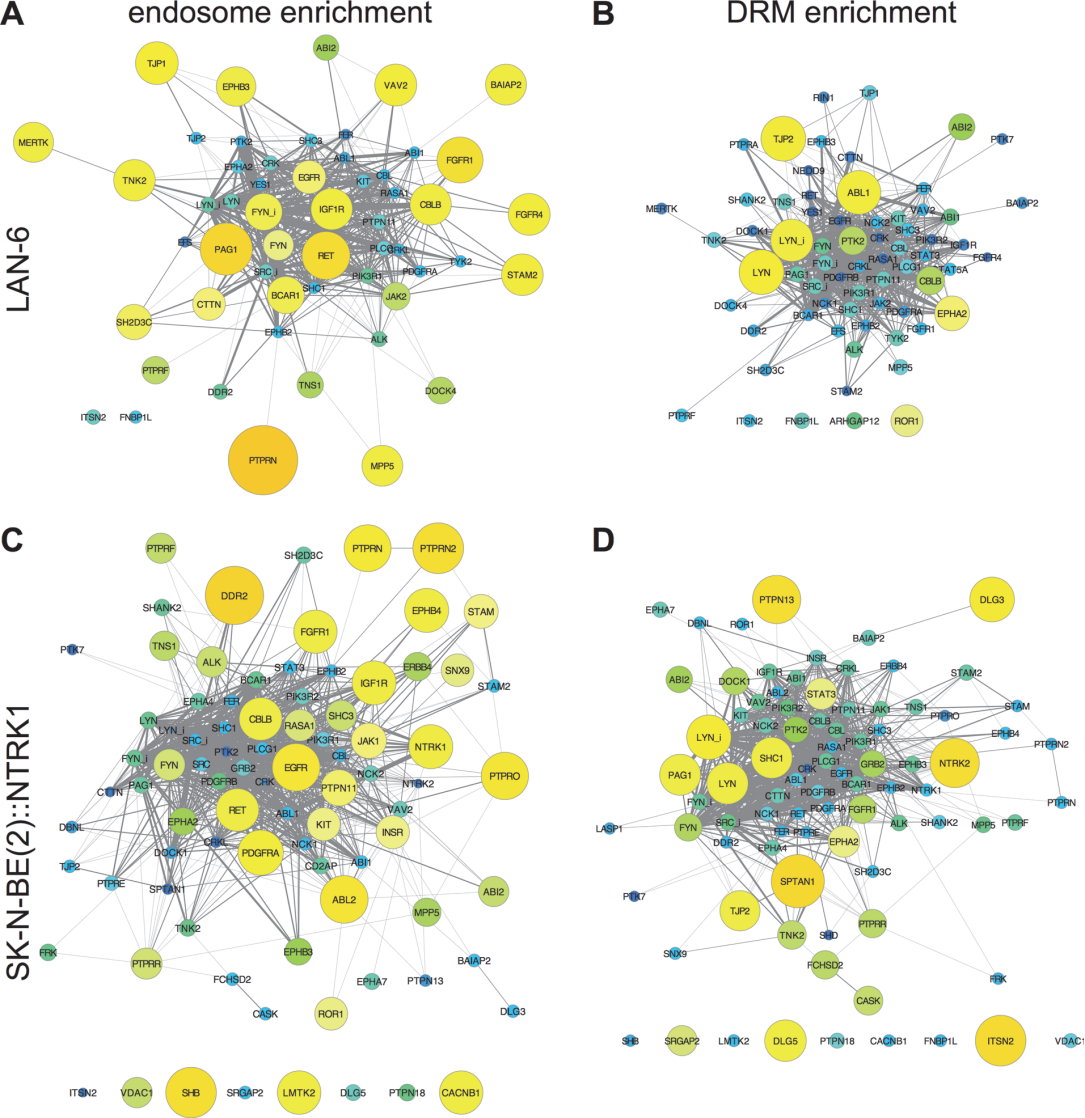
Enrichment of RTKs in endosomes and DRM fractions. Enrichment was graphed in PPI networks as big yellow nodes for positive enrichment and small blue nodes for de-enrichment. Green nodes of intermediate size indicate equal amounts in all fractions. PNCPs from LAN-6 endosomes (**A**) and DRMs (**B**); SK-N-BE(2) expressing TrkA (NTRK1) endosomes (**C**) and DRMs (**D**).

We asked whether particular phosphorylated proteins were enriched in endosomes and DRMs by calculating the ratio between amounts in those fractions compared to proteins in all other samples from the same cell line. ALK, FGFR1, RET, PDGFRA, DDR2, EGFR, and IGF1R were enriched in endosomes from two or more neuroblastoma cell lines, but there were profound differences among cell lines (Fig 5B). In Fig 6, enrichment was graphed in PPI networks as big yellow nodes for positive enrichment and small blue nodes for de-enrichment (defined as lower amounts in that fraction compared to elsewhere). In LAN-6 cells, most RTKs were enriched in endosomes, except EPHA2 and ROR1, which were enriched in DRMs (Fig 6A and 6B). In SK-N-BE(2) cells made to over-express NTRK1/TrkA, this receptor was enriched in endosomes and de-enriched in DRMs, whereas its related receptor, NTRK2/TrkB, had the opposite pattern, being enriched in DRMs and de-enriched in endosomes (Fig 6C and 6D). The SFKs, FYN and LYN were localized differently, with LYN (and LYN_i) being enriched in DRMs in LAN-6 and SK-N-BE(2) cells, and FYN (and FYN_i) being enriched in endosomes in LAN-6 cells, but not in SK-N-BE(2) cells (Fig 6). PAG1 was enriched in endosomes in LAN-6 cells (Fig 6A) and, in contrast, in DRMs in SK-N-BE(2) cells (Fig 6D).

We noted differences in distribution of SFK and PAG1 phosphorylation on individual phosphorylation sites between the two cell lines (Fig 5C). For example, PAG1 81 was consistently phosphorylated in endosomes, and PAG1 317 was consistently phosphorylated in DRMs in both cell lines, yet PAG 359 and other sites were highly phosphorylated in LAN-6, but not SK-N-BE(2) endosomes (Fig 5C). These data suggest a relationship between SFK and PAG1 phosphorylation on specific sites and intracellular localization.

### FYN and LYN Changed Intracellular Location upon RTK Stimulation

**Fig 7:**
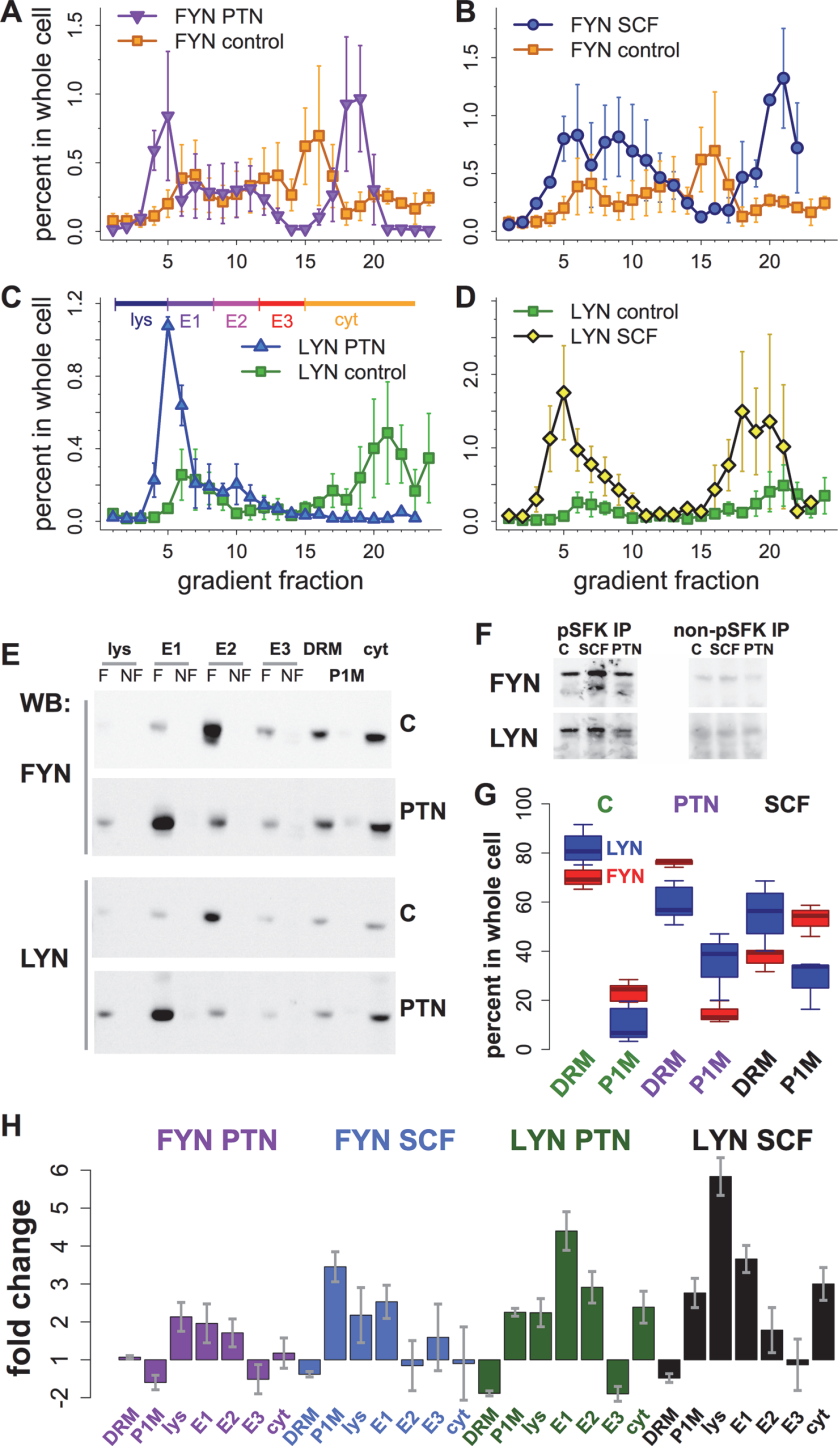
Intracellular localization of FYN and LYN changed in response to PTN and SCF. (**A-D**) Velocity gradient fractionation of intracellular organelles after serum starvation (control; squares) or 60 min stimulation of LAN-6 cells with PTN (**A, C**) or SCF (**B, D**). Data were quantified from western blots using antibodies against FYN (**A, B**) and LYN (**C, D**) and expressed as the percent of each protein in each gradient fraction after quantifying amounts in all other cell fractions (percent in whole cell). Shown are means from 2–4 experiments for each condition; error bars are SEM. (**E**) Organelle fractions, defined as pools of velocity gradient fractions lys, E1, E2, E3, and cyt as shown in (**C**), were subjected to flotation equilibrium centrifugation [32]. Western blots show these fractions and detergent-resistant (DRM) and-soluble (P1M) fractions (see S10 Fig) after no treatment (C = control) or treatment with PTN, probed with antibodies to FYN and LYN (indicated). Both floating (F) and non-floating (NF, defined as material in higher density fractions at the bottom of flotation equilibrium gradients) membranes were analyzed. That SFKs associated with floating (F) fractions indicates that they were robustly bound to membranes. (**F**) Phospho-SFK (left) and non-phospho-SFK antibodies (right) were used to immunoprecipitate proteins from endosome E1 fractions under unstimulated or stimulated conditions as in (**A**). Western blots were probed with FYN- and LYN-specific antibodies (indicated). (**G**) Box plot shows amounts of FYN (red) and LYN (blue) in detergent-resistant (DRM) and-soluble (P1M) fractions under control, ALK- or KIT-stimulated conditions as in **A**-**D**. (**H**) Bar plots show fold change (treatment/control if positive;-(treatment/control)^-1^ if negative) in all cell fractions under unstimulated or stimulated conditions as in **A**-**D**. (**G, H**) Amounts of FYN and LYN in all cell fractions were quantified from 3–7 experiments; boxes show quartiles and whiskers show ranges in **G**, error bars are SEM in **H**.

These data suggest the hypothesis that stimulation of different RTKs should affect the activity and intracellular localization of FYN and LYN. We used a cell fractionation approach to assay intracellular localization after stimulation of ALK with PTN and KIT with SCF (Fig 7). Amounts of FYN and LYN increased with PTN and SCF treatment in organelles whose migration on velocity sedimentation gradients overlaps with Rab7 and acid phosphatase [32], markers for late endosomes and lysosomes (Fig 7A–7D, fractions 4–7). SCF also induced increases mainly in FYN localization to fractions 8–11 (Fig 7B and 7D), which contain endosomes marked by Rab4 and Rab5 [32]. LYN and FYN also increased in fractions 16–22 in response to both ligands (Fig 7A–7D). These fractions contain soluble, cytoplasmic proteins, and signaling particles, which were previously resolved on gradients centrifuged with greater force [52]. FYN and LYN were robustly associated with membranes that floated to the density of endosomes on floatation equilibrium gradients, and amounts increased in organelles of higher sedimentation velocity (E1) after PTN treatment (Fig 7E). Both FYN and LYN were predominantly phosphorylated on their activating sites in these membranes (Fig 7F). Differences between FYN and LYN localization to detergent-resistant and-soluble fractions were also observed. FYN’s response to PTN (enhanced DRM and diminished P1M association; Fig 7G) was different from that to SCF (reduced DRM, enhanced P1M association). In contrast, LYN’s response to both ligands was similar (reduced DRM, increased P1M association; Fig 7G). The magnitude of ligand-induced changes in FYN and LYN in organelle fractions were distinct in response to PTN and SCF (Fig 7H). Increased FYN and LYN in faster sedimenting organelles (lys and E1 fractions) likely reflects migration to multivesicular bodies, late endosomes, and possibly lysosomes [32]. These data are consistent with the hypothesis that RTK activation regulates FYN and LYN localization and activity in neuroblastoma cells in a manner that distinguishes responses to individual RTKs.

### Phosphorylation Site Clusters

**Fig 8:**
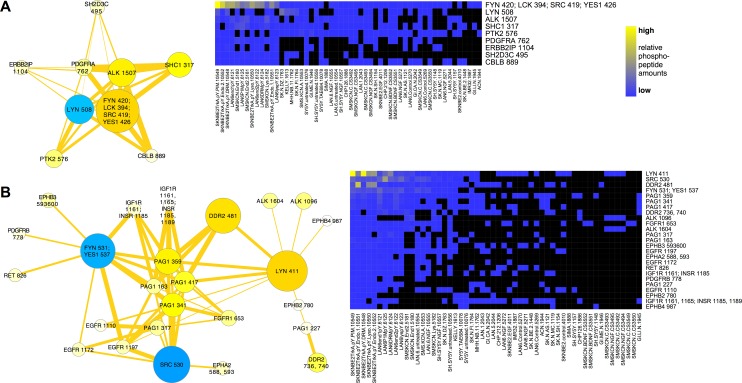
Phosphorylation site clusters displayed as a co-clustered correlation networks (CCCNs). RTK, SFK and PAG1 phosphorylation sites were selected from the co-cluster correlation network shown in S11 Fig, where edges represent Spearman correlations greater than 0.5 from phosphorylation sites that co-clustered from t-SNE embeddings. Edge line thickness is proportional to correlation and the number of cases in which phosphorylation sites co-clustered from different embeddings. Two separate groups containing the most highly phosphorylated RTK and SFK sites are shown. Heat maps (right) show the primary data for phosphorylation on specific sites. (**A**) The network containing phosphorylation sites ALK 1507 (the most highly phosphorylated site on ALK), FYN 420, PDGFRA 762 and LYN 508 was extended to include other PNCP phosphorylation sites that co-clustered with positive Spearman correlation; no PAG1 phosphorylation sites co-clustered with this group. (**B**) Five highly phosphorylated PAG1 sites co-clustered with phosphorylation sites on IGF1R, DDR2, EGFR, and FGFR1. Note that the conserved peptide sequence for FYN 531; SRC 530; YES1 537 was inclusively summed to both FYN 531; YES1 537 and SRC 530 in the phosphorylation site network, but exclusively summed to FYN_i in the total protein phosphorylation networks based on the presence of other FYN phosphopeptides in the same samples. By the exclusive criteria, FYN 420; LCK 394; SRC 419; YES1 426 in (A) most likely represents FYN activation; FYN 531; YES1 537 most likely represents FYN inhibitory phosphorylation; and the inclusive summing method likely over-represents the amounts of SRC inhibitory phosphorylation in (B).

These data motivated further higher resolution interrogation of the relationships between individual protein phosphorylation events. We investigated the relationships among phosphorylation sites by clustering phosphorylation sites (summed from homologous phosphopeptides) and visualizing data structure as a co-cluster correlation network (CCCN). The edge-weighted, spring-embedded layout of this network showed several distinct groups of sites with statistical relationships to other groups (S11 Fig). The data were interrogated with a focus on the most highly phosphorylated sites on RTKs, SFKs, and PAG1 to ask if phosphorylation sites cluster together. Two distinct clusters are shown in Fig 8. ALK was detected in 22 distinct phosphopeptides in neuroblastoma samples, which could be collapsed into 13 distinct phosphorylation sites based on sequence homology. Fig 8A shows that the ALK phosphorylation site, ALK 1507, which was most frequently seen in neuroblastoma samples, was associated with inhibited LYN (LYN 508), and activated FYN (FYN 420; LCK 394; SRC 419; YES1 426; this site was assigned to FYN in total phosphorylation calculations because other FYN phosphopeptides were detected in the same samples; see Materials and Methods). Co-clustered phosphorylation sites on several other proteins in this cluster resemble the cluster in Fig 1A. Fig 8B shows that other ALK phosphorylation sites (ALK 1096 and 1604) clustered with the most prominently detected phosphorylation site on DDR2 (DDR2 481), along with activated LYN (LYN 411), and inhibited FYN and SRC (FYN 531; YES1 537 and SRC 530). Also co-clustered with the group in Fig 8B were phosphorylation sites from other RTKs represented in the cluster in Fig 1B.

Strikingly, a number of phosphorylation sites on PAG1 were detected, but none were statistically clustered with activated FYN and inhibited LYN (Fig 8A), while the most prominent PAG1 phosphorylation sites clustered with activated LYN and inhibited FYN and SRC (Fig 8B). The data suggest a mutually antagonistic relationship between different SFKs, particularly LYN and FYN, so that when one is activated, the other is inhibited. Phosphorylation of PAG1, which recruits SFKs and their inhibitory kinase, CSK, to bind to it [51], appears to be associated with the state where LYN is activated, and FYN and other SFKs are inhibited (Fig 8B).

The data suggest that RTK phosphorylation does not occur on all sites at once under all conditions, resulting in different phosphorylation sites on ALK and other RTKs clustering separately from one another. RTKs phosphorylated on different sites also fractionated to endosomes and DRMs selectively (S12 Fig). For example, some ALK and KIT phosphorylation sites were enriched in endosomes, while others were enriched in DRMs, with differences between the two cell lines examined (S12 Fig). In contrast, all EGFR and RET phosphopeptides were consistently enriched in endosomes. Phosphorylation on selected sites would be consistent with RTKs acting as effectors as well as initiators of signal transduction. Phosphorylation by other tyrosine kinases, such as other RTKs or SFKs, may favor particular sites, and thus influence intracellular location, providing different contexts for signaling pathways to influence cell responses.

## Discussion

### Approach

There is considerable interest in tyrosine kinase signaling mechanisms because of their roles in tumor initiation and metastasis. Tyrosine kinase signaling mechanisms arose during evolution when multicellular organisms evolved [19,54], and many RTKs are known to be involved in governing cell behaviors such as cell division, cell death, differentiation, and migration. Acquisition of phosphoproteomic data from a migratory, multipotent tumor cell type was motivated by these considerations. The complexity of the data forced us to develop new approaches to understand signaling mechanisms that involve tyrosine phosphorylation. Indeed, modeling dynamic complex systems and their interacting macromolecules remains a general challenge that lags far behind large-scale acquisition of biological data [35,55]. To make progress, we found it useful to apply techniques from the fields of pattern recognition and graph (network) theory and combine them with external PPI and GO data [34], thus extending the concept of using a variety of statistical techniques for exploratory data analysis [56]. Exploratory data analysis is inherently descriptive in its initial stages, but allows generation of hypotheses which then motivates more directed data interrogation and subsequent experiments. In this way, initial phosphoproteomic analysis of neuroblastoma cell lines motivated further experiments where cells were treated to perturb signaling pathways and subjected to organelle fractionation.

Several technical hurdles had to be overcome when analyzing these data. 1) Phosphoproteomic data, like any mass spectrometry data, has missing values because many peptides are not analyzed by the detector, and using a “data not available” marker (NA) instead of zero facilitated calculation of statistical relationships based only on observed data [34]. 2) Employing an effective embedding technique [48,49] prior to clustering allowed resolution of patterns that were difficult to discern otherwise [34]. 3) The analysis treated all samples mathematically as different states in the total neuroblastoma signaling system. Embeddings were first performed on data from cell lysates from 21 neuroblastoma cell lines grown in culture without treatments. Preliminary analysis led to emerging trends in clustering that resembled the more robustly defined clusters derived when all samples were included, including different cell lines, cells treated in ways to perturb signaling pathways, and cell fractions. In fact, we noted different phosphoproteomic results from the same cell lines cultured under nominally similar conditions when grown by different investigators or at different times. This heterogeneity could be due to differences in serum batches, selection pressure by passaging, or other factors. Mathematically, any heterogeneity is useful for statistical analyses because different phosphorylation patterns help distinguish signaling pathways. 4) Visualizing data as networks was informative in several ways. Initially, networks that included all PPI edges allowed us to determine that the dataset was complete enough for further analysis (S1 and S2 Figs). However the multitude of known interactions among signaling proteins was too complex to be informative, and not specific to neuroblastoma.

Importantly, to visualize data structure in a new and informative way, we developed an innovation that may be generally useful, namely to filter edges to show interactions only between co-clustered components (CFN, Fig 3; CCCN, S7 and S11 Figs). Including clusters from different (equally valid) embeddings recognizes that tyrosine kinase signaling pathways are highly interconnected by conveniently allowing overlap in cluster membership. Applying this approach to individual phosphorylation sites (S11 Fig) elucidated phosphorylation patterns and relationships among signaling pathways with high resolution. These graphs allowed exploration of data structure using network analysis in a visually accessible graph. We focussed here on PNCPs (proteins with tyrosine kinase, tyrosine phosphatase, SH2 and SH3 domains). Cytoscape-accessible files of these graphs are provided online for investigators interested in exploring the data further S2 Dataset.

### Compartmentalization and Collaboration in RTK Signaling Pathways

The observation that neuroblastoma cell lines expressed more than half of the RTKs in the human genome (S3 Fig), and responded to signals from growth factors in the embryonic microenvironment to migrate and differentiate into a number of neural crest target sites (S4 Fig), suggests that neuroblastoma, and neural crest from which it is derived, takes full advantage of RTK signaling mechanisms to govern cell fate decisions. We found functional compartmentalization of tyrosine kinase signaling pathways in neuroblastoma cells from different tumor origins, with different sets of RTKs forming collaborative groups that interact with each other and common downstream effectors (Fig 3). There was also physical compartmentalization of signaling components within neuroblastoma cells. By combining cell fractionation with phosphoproteomics, we found that there was non-uniform distribution of signaling components, and moreover non-uniform distribution of phosphorylated residues on individual proteins (Figs 5, 6 and S12 Fig).

Compartmentalization of signal-initiating receptors and downstream effectors may be employed to distinguish extracellular instructions that determine cell fate. Many receptors signal from endosomes to amplify signals, activate different effectors than those activated at the plasma membrane, or convey signals to different intracellular locations [30,57–61]. In fact, there is evidence that endosomal signaling from a number of different receptors affects cell fate decisions during development [62–66].

Different RTKs elicit different cellular responses, yet all appear to activate the canonical RAS/ERK, PLC-γ, SFK, and PI3K/AKT pathways. Differential responses may be obtained by affecting the duration of downstream effector activation [67], or by modulating the relative strength of downstream pathway signaling, as has been elegantly shown for the ratio of activation of AKT and ERK pathways that distinguishes the proliferation and neurite-outgrowth (differentiation) response in PC12 cells [68]. Our data suggest that SFKs, especially FYN and LYN, function as signal integrating devices—central hubs in the tyrosine kinase signaling network—to distinguish RTK signal transduction pathways, in part by activating distinct mechanisms specifically in endosomes and lipid rafts. FYN and LYN were highly phosphorylated in endosomes and detergent resistant membranes, and their activity and localization was affected by cell type (Figs 5 and 6) and changed in different ways in response to receptor activation (Fig 7). FYN and LYN appear to have a partially antagonistic relationship because when one is activated, the other is frequently phosphorylated on its C-terminal inhibitory site (Figs 1 and 8). How localized activation of FYN and LYN may in turn affect the relative strength and duration of effector pathways, or the ratio of activation of AKT and ERK, remains to be determined.

Previous work supports the hypothesis that SFKs function to affect signal integration and protein localization. SFK family members are differentially palmitoylated, which affects their localization on endosomes and the plasma membrane [69,70]. SFKs have been implicated in the regulation of endocytosis by a variety of mechanisms. These include phosphorylation of clathrin [71], modification of Rho proteins and actin assembly [69,72], and regulation of the Cbl family of ubiquitin ligases [51,73,74], which control RTK sorting in endosomes [75,76]. The localization of SFKs to lipid rafts is thought to be important for their signaling function [77]. For example, it has been shown that FYN plays a role in localizing NTRK2/TrkB to lipid rafts [78], and LYN, which is enriched in lipid rafts (Fig 6) is a key effector of NTRK1/TrkA for terminal differentiation [79].

The transmembrane SFK scaffold protein PAG1 (Cbp/PAG) has been previously described to associate with lipid rafts [80]. Consistent with this, we found PAG1 in DRMs (Figs 5 and 6). PAG1 was also one of the most highly phosphorylated proteins in endosomes (Figs 5 and 6). PAG1 binds several different SFK family members, and can bind to more than one at a time, as well as to the kinase that phosphorylates the inhibitory site on them, CSK [51]. In fact, PAG1 can form a complex with a number of SFK regulatory proteins in addition to CSK: the phosphatase, PEP, PTPN22 and SOCS1, which catalyses SFK ubiqutination [51]. PAG1 also binds PLCG1/PLCγ1 and PI 3-kinase; and PLCG1 and PIK3R1/2 were detected in endosome fractions in this study. The phosphatase, PTPN11/SHP-2, which was also prominently detected in neuroblastoma endosomes (Fig 5A) may also be part of this complex [81]. Different patterns of PAG1 and PTPN11 phosphorylation in leukemia and prostate cancer are associated with different activation states of SFKs and other signaling effectors [82,83]. This array of proteins bound to the PAG1 scaffold may either positively or negatively regulate SFK activity as well as other effectors, depending on context. Interestingly, we found phosphorylated PAG1 to be clustered with activated LYN and inhibited FYN (and SRC), but not activated FYN and inhibited LYN (Fig 8).

The collaborative groups that emerged from these data (Fig 3) suggest the hypothesis that receptors within these groups might be likely to cause transactivation of other RTKs within the same group. IGFR1 1161 phosphorylation was decreased by the ALK inhibitor on a similar scale to ALK 1507 and 1509 (S9 Fig), which is consistent with the hypothesis that ALK and IGFR1 activities are linked (Fig 3A). The data show substantial variability on different RTK phosphorylation sites, however. When we performed clustering on individual phosphorylation sites (S1 Fig), different phosphorylation sites on ALK and other RTKs clustered separately from one another. For example, ALK 1507 was associated with the group of sites shown in Fig 8A, while ALK 1096 and 1604 was associated with the group in Fig 8B. These phosphorylation patterns may be due to selective phosphorylation or dephosphorylation. Phosphorylation on selected sites would be consistent with RTKs acting as effectors as well as initiators of signal transduction; other tyrosine kinases, such as other RTKs or SFKs, may favor phosphorylation on particular sites. One mechanism of RTK transactivation could involve heterodimerization of different RTKs or multiprotein receptor clusters. Heterodimers have been inferred from co-immunoprecipitation between MET, EGFR, and ERBB3/Her3 [84]; PDGFR and EGFR [85]; AXL and EGFR [86]; and among similar EGFR and FGFR family members [87].

SFKs may also play a role in RTK transactivation [87]. SFK SH2 domains bind to phosphorylated tyrosine residues on RTKs, and can phosphorylate RTKs directly, in some cases mimicking those sites phosphorylated during ligand-induced receptor activation [74]. SFKs associate with RTKs in protein complexes and play a direct role in transducing their signals [74,88]. It has been shown that transactivation between PDGFR and EGFR depends on SFKs [85], and SRC is recruited to PDGFRB and the GPCR, MBTPS1/S1P1, which form a complex that is endocytosed as a unit [89]. In addition, phosphatases may favor particular sites. For example, the phosphatase, PTPN6/SHP-1, acts on NTRK1/TrkA, mainly at Y674 and Y675 [90], and association of PTPN6/SHP-1 with lipid rafts suggests localized dephosphorylation of NTRK1/TrkA [33].

### RTK Pathways in Neuroblastoma and Neural Crest

Point mutations in the RTK, ALK, are the primary cause of familial neuroblastoma and account for 8–12% of sporadic neuroblastomas [15]. ALK is expressed earlier than Trks (NTRK1-3) in neural crest development [91], highly expressed in paravertebral sympathetic ganglia [92], and co-expressed with NTRK1/TrkA and RET in a subtype of dorsal root ganglia neurons during development [93]. Overexpression of full-length ALK in PC12 cells causes increased phosphorylation of PTPN11/SHP-2 and STAT3 [94]. We found PTPN11 clustered with ALK and IGF1R (Figs 1B and 3A), and localized in endosomes (Fig 5A). STAT3 co-clustered with ALK, IGF1R and PDGFRA as part of the same collaborative group (Fig 3A). That phosphorylated ALK was present in many neuroblastoma cell lines is consistent with its role as an important marker, or driver, of neuroblastoma.

Like ALK, KIT is an emerging marker for aggressive neuroblastoma that leads to poor prognosis [95]. We found KIT in a subset of neuroblastoma cell lines, and enriched in endosomes in SK-N-BE(2) cells (Figs 6 and S6C). Activation of either ALK or KIT caused increased association of FYN and LYN with endosomes (Fig 7). Both ALK and KIT are expressed early in neural crest, giving rise to the hypothesis that cells derived from an earlier stage of the neural crest sympathoadrenal lineage are more likely to give rise to more aggressive tumors and poor clinical outcome. Sox10+/Kit+, but not Sox10+/Kit- cells, remain multipotent even after reaching their final target tissue [96,97]. Neuroblastoma cells that express high levels of KIT can induce tumors ninefold more efficiently than those with low KIT expression [95]. Interestingly, KIT clustered with ROR1 (S6C Fig), which is also expressed early in development and is a marker for cell migration and invasiveness in neuroblastoma and other cancers [98]. The data suggest that both KIT and ALK may be active early in neural crest development and their activity signifies, or causes, incomplete differentiation.

Neurotrophin receptors are of interest in neuroblastoma because they are markers for clinical prognosis. NTRK1/TrkA is a marker for neuroblastoma tumors that spontaneously undergo apoptosis and regression, while NTRK2/TrkB is often expressed with its ligand, (BDNF), forming an autocrine loop that predicts poor prognosis [99–101]. The pan-neurotrophin receptor, p75^NTR^ enhances sensitivity to low neurotrophin levels, which affects response and outcome in NTRK1/2-expressing cells [102]. Overexpression of NTRK1/TrkA in LAN-6 cells caused apoptosis, but was tolerated in SK-N-BE(2) neuroblastoma cells that express non-functional p53, in agreement with previous work [103]. The differential localization of NTRK1/TrkA, which preferred endosomes, and NTRK2/TrkB, which was enriched in DRMs (Fig 6C and 6D) may provide a clue as to how these two similar receptors have such profoundly different effects in neuroblastoma. Neurotrophin receptors signaling from lipid rafts vs. endosomes may account for the selectivity of their transduced signals and the resulting effects on cell behavior [33,104,105].

### Conclusion

Neuroblastoma cell lines offer insight into neural crest signaling pathways that is difficult to obtain directly from migrating immature neural crest cells. While signaling pathways activated by oncogenic mechanisms and cell culture conditions no doubt contribute to the phosphorylation patterns we identified here, the fact that these cells retained the capacity to migrate and differentiate (S4 Fig) indicates that neuroblastoma cell lines retain signaling pathways activated in immature, multipotent neural crest [2,4,7,8]. That neuroblastoma cells express so many RTKs suggests that mechanisms to discern and integrate different receptors’ signals must play a role in cell fate decisions in neural crest and neuroblastoma [106–108]. SFKs, which contain a tyrosine kinase domain, a SH2 domain that recognizes phosphorylated tyrosine, and a SH3 domain that plays a conserved role in endocytosis (and other) mechanisms, appear to be constructed for signal integration. The activation and dynamic intracellular location of LYN and FYN, and a scaffold protein (PAG1) that binds to them, suggest that these SFKs function to discern and integrate signals from different RTKs.

Discovery of new pathways activated in neuroblastoma cells provides potentially new therapeutic approaches [109]. Co-activation of two or more RTKs, which is not uncommon in cancer, leads to therapeutic challenges that compel consideration of treatment with multiple inhibitors [110–113]. The data and analysis presented here suggest, for example, that ALK-driven tumors might also present activated IGF1R, FGF1R, and/or PDGFRA. When challenged by ALK inhibitor therapy, these receptors could take over as drivers to activate similar signaling pathways (Fig 3A). The data also suggest that there are different routes to cell proliferation in neuroblastoma, such as the distinct mechanisms activated by the EGFR group (Fig 3B), or KIT (Figs S6C and 7). In the future, it will be important to compare our results to pathways activated in neuroblastoma primary tumors in different microenvironments. This study, and other large-scale gene expression or proteomic studies that include network and pathway analysis [82,83,114] and gene ontology [115], will help determine likely control points for cell growth, migration, and differentiation in individual tumors.

## Materials and Methods

### Cell Treatments and Fractionation

21 neuroblastoma cell lines were obtained by Cell Signaling Technology (Danvers, MA) from American Type Culture Collection (ATCC; Manassas, VA); Leibniz Institute DSMZ-German Collection of Microorganisms and Cell Cultures (DSMZ; Braunschweig Germany); Coriell Institute for Medical Research (Camden, NJ); and Interlab Cell Line Collection (ICLC; Genova, Italy). SMS-KCN, LAN6, SK-N-BE(2), and SH-SY5Y, were obtained by M.G. from Children’s Hospital of Los Angeles, CA, except for SH-SY5Y cells, which were provided by Dr. Mark Israel (University of California, San Francisco, CA). Neuroblastoma cells were grown in RPMI 1640 medium (Thermo Scientific HyClone, U.S.) supplemented with NaHCO_3_ (Sigma, U.S.) and 10% fetal bovine serum (Thermo Scientific HyClone, U.S.).

Tyrosine phosphoproteomic data for 21 neuroblastoma cell lines were initially acquired at Cell Signaling Technology using techniques described previously [41,42]. Cells were incubated overnight in media without serum prior to harvesting for mass spectrometry. Four cell lines [SH-SY5Y, LAN-6, SMS-KCN, and SK-N-BE(2)] were selected for further studies because of different point mutations in ALK, p53 status, RTK expression, morphology, and growth characteristics. A sub-line of adherent SMS-KCN cells, named SMS-KCN-A, was selected by culturing SMS-KCN cells on collagen coated plates and removing floating cell spheres. SMS-KCN-A cells required trypsin for passage and retained their adherent phenotype after passaging. SK-N-BE(2) cells were made to overexpress Rat TrkA with CFP insert at amino acid 587 (in the cytoplasmic tail) using a γ-retroviral expression vector (a gift from Mary Beth Eiden, NIH [116]). The construct was made using transposon-mediated insertion [117], and shown to be functional as assayed by NGF-induced tyrosine phosphorylation and neurite outgrowth in PC12^nnr5^ cells (in which endogenous TrkA is non-functional). The γ-retroviral genomic vector plasmid (pRT43.2TrkCFP), helper plasmids (pIK6.1.gagpol+ATG and pLP-VSVG) were transfected into HEK293T cells using calcium phosphate. Cell culture media (Dulbecco’s Modified Eagle Medium/10% FBS) was changed approximately 16 hours post-transfection. Supernatant containing viral particles was harvested at 48 and 72 hours post-transfection and pooled together.

Cell lines were treated (or left untreated, control) with ligands or the ALK inhibitor TAE684 as indicated in Table 1.

**Table 1:** Neuroblastoma Cell Line Treatments.

**cell line**	**ALK mutation**	other phenotypes	**treatments**
LAN-6	ALK D1091N	slow growing, neuronal	NGF (2 nM), 10 min
SMS-KCN	ALK R1275Q	form neurospheres, secrete BDNF	BDNF (2 nM) 10 min
			NGF (2 nM) 10 min
SK-N-BE(2)	ALK (wild type)	very fast growing, N-myc amplified, p53^–^	EGF (2 nM) 10 min
SH-SY5Y	ALK F1174L	fast growing	NGF (2 nM) 10 min
			TAE684 (200 nM) 6 h

For organelle fractionation phosphoproteomics, cell lines were treated with ligands (LAN-6 and TrkA-CFP-expressing SK-N-BE(2): NGF, SMS-KCN:BDNF) for 10 min at 37°C. For cell fractionation experiments after ALK and KIT stimulation (Fig 7), LAN-6 cells were serum-starved for 2hr, then treated with 50 nM PTN or 5 nM SCF (R & D Systems). Ligands were bound to cells at 4°C for 1 hr, then cells were warmed to 37°C for 10 min or 1 hr. Organelles were isolated from mechanically permeabilized cells using velocity sedimentation only (Fig 7A–7D) or velocity sedimentation followed by flotation equilibrium centrifugation as described [32]. Phosphoproteomic analysis was performed on two endosome (E1, E2) and lysosome (Lys) fractions as shown in S10 Fig mass-density plots. In addition, E3 and cytosol (cyt) fractions collected from velocity gradients as indicated in Fig 7C were methanol/chloroform precipitated for gel electrophoresis and western blot analysis. Detergent-resistant (DRM) and-soluble (P1M) fractions were prepared as described [33] except that flotation of detergent-resistant membranes was not performed for mass spectrometry experiments or gel electrophoresis and western blotting.

Antibodies used in Fig 7 were from Cell Signaling Technologies (Danvers, MA): anti-LYN (#2796);-FYN(#4023);-pSRC (Y416; #2101);-non-pSRC (Y416; #2102). HRP-linked secondary antibodies were from GE Healthcare UK Limted: anti-Rabbit HRP (# NA934V); anti-Mouse HRP (#NA931V). Ligands were from R & D Systems: PTN (#252-PL); SCF (#255-SC).

### Phosphoproteomics

Quantification of immunoprecipitated phosphopeptides was obtained from the peak intensity of each peptide (from the MS1 spectrum of the intact peptide before fragmentation for MS/MS analysis) [41,42]. Data were processed using R [118,119]. Gene names were mapped and converted to unique gene identifier names (according to genenames.org). In cases where conserved peptide sequences identified multiple proteins, if a protein was identified by a different peptide in the sample, the peptide was assigned to that protein, otherwise the first name was used (this is referred to as exclusively summed). Where phosphorylation sites were known to have inhibitory effect on protein activity (Regulatory_sites.gz), peak intensity values were converted to negative values (this allows graphing network nodes as blue, as in Fig 1). Peak intensity was summed for each protein in each sample (i.e., cell line) using functions written in R [34], except in the case of SRC-family kinases (SFKs), where peptides phosphorylated on C-terminal inhibitory sites were tracked separately (denoted FYN_i, LYN_i, SRC_i, YES1_i, FRK_i). Due to limits in mass spectrometry detection, data were not expected to be complete; for example SMS-KCN cells express NTRK2 (TrkB), but NTRK2 peptides were masked; and NTRK1 was not always detected in cell lines known to express it. Therefore, missing values were treated as NA (data not available) for statistical calculations [34].

In cases where duplicate mass spectrometry analyses were conducted on the same cell line, under the same conditions within a short time frame (*e*.*g*., duplicate runs of the same experiment), data were merged to include the average of the two runs, ignoring missing values. Otherwise, each experiment was treated as an independent sample for data analysis.

Summarized data are available online as Supplemental Data (*S3 Dataset*). Primary phosphoproteomics data are available from PhosphoSitePlus (http://www.phosphosite.org/browseDiseaseResultAction.do?id=66&type=true) using curation set (CS) numbers 1119, 1121, 1148, 1154, 1157, 1206, 1448, 1613, 1762, 1763, 1764, 1886, 1887, 1888, 2042, 2043, 2044, 3492, 3493, 3495, 4010, 4011, 5180, 5181, 5182, 5269, 5270, 5271, 5272, 6121, 6122, 6123, 6124, 6125, 6151, 6152, 6153, 6154, 6155, 9206, 9208, 9942, 9943, 9944, 9946, 10553, 10554, 10555, and 10557. Supplementary data, and links to spectra and other important experimental parameters from this submission will be also made available via the ‘Reprints, References, Supplemental Tables’ page http://www.phosphosite.org/staticSupp.do.

Duplicate MS runs on the same samples in the same experiment were LAN6.Control (CS 5269, 5270); LAN6.NGF (CS 5271, 5272); SMSKCN.Control (CS 3492, 3493); SMSKCN.NGF (CS 3494, 3495); SMSKCN.Control (CS 3549, 3550); SMSKCN.BDNF (CS 3551, 3552).

### Data Analysis and Clustering

1203 tyrosine phosphorylated and 557 AKT-substrate (using RxRxxS/T consensus sequence antibodies) proteins were identified in all samples; 138 were in common between phosphotyrosine and phospho-AKT substrate data.

For analysis of proteins, the total amount of phosphorylation of each protein was determined by summing peak intensity signals from all peptides for each protein in each sample. In cases where conserved sequences did not allow unambiguous assignment to a particular protein, peptides were assigned to proteins that were detected by other phosphopeptides in the same sample or the first name was used. We thus obtained a data matrix in which each row corresponds to a protein and each column corresponds to a neuroblastoma cell line or organelle fraction (i.e. a sample; Fig 1). The elements of the data matrix contain the total peak intensity signals. All samples were treated as different states in the neuroblastoma system. To ensure that all samples were weighted equally in statistical calculations, data were normalized by scaling by sample standard deviations without centering. The statistical similarity of any two proteins was determined by the extent to which they were detected in similar amounts in each sample. This relationship was represented in different ways. First, the Euclidean distance between the row vectors corresponding to the two proteins was stored in a distance matrix. A dissimilarity matrix (also called dissimilarity representation or feature vector) is similar to a distance matrix except the values do not necessarily specify Euclidean distance [120,121]. For the second method, dissimilarity was represented by one minus the absolute value of the Spearman correlation of each protein with every other protein. A third method employed combining equally scaled Euclidean distance and Spearman dissimilarity as a dissimilarity matrix, referred to as Spearman-Euclidean dissimilarity, or SED [34,122].

The dimension-reduction (embedding) technique, t-distributed stochastic neighbor embedding (t-SNE) was employed to visualize the proteins in a scatter plot based on the distance or dissimilarity matrices [34,48,123]. This machine learning technique aims to represent each protein by a two-(or three-) dimensional point, arranging the points in such a way that nearby points in the scatter plot correspond to proteins with statistical similarity and distant points to dissimilar proteins. Proteins close to one another in this data structure were identified as clusters by the minimum spanning tree, single linkage method [50]. Three dimensional embeddings of data structure were visualized with PyMOL and Cytoscape, the latter using RCytoscape and three dimensional manipulation functions from the R package, *rgl* (S1 Movie). Filters were applied to focus on proteins containing tyrosine kinase, tyrosine phosphatase, SH2, and SH3 domains (PNCPs), or to focus on proteins that clustered together using both Spearman and Euclidean dissimilarity embeddings [34].

Clusters were evaluated by several quantitative measures. For comparison, 70 non-overlapping random clusters were generated containing gene names from the data set; the number of members was also randomized to mimic the number of genes identified in clusters defined by t-SNE embedding and minimum spanning tree methods. Evaluations based on examining the primary data (internal evaluations) were performed as described [34]. A quantitative index was used to evaluate the density of data (percent NA or missing values) and the conformity to the pattern in the group, weighted by the total phosphopeptide signal (S5A and S5B Fig). External evaluations with data from PPI (S5C and S5D Fig) and GO (S5E and S5F Fig) databases were also compared to 20 random clusters [34].

PPI edges from String (string.embl.de/) [124], GeneMANIA (genemania.org/) [125], and the kinase-substrate interactions from PhosphoSitePlus (phosphosite.org) [126] were merged as described [34]. Network modules or highly interconnected regions of the neuroblastoma phosphoproteomic network (S2B Fig) were determined using Cytoscape plugins MCODE and NeMo.

Gene Ontology (GO) was determined as described [34]. Enriched gene function annotations, or GO terms for gene groups determined by clustering methods, and for the randomly selected genes as described above, were retrieved using Bioconductor libraries “GO.db,” “GOstats,” and “org.Hs.eg.db” ([127] bioconductor.org/) using a p-value <0.01. If there was enrichment, at least two genes in the cluster should have the same GO term, so terms with single genes were discarded. The enriched GO terms per gene was compared to the average background for randomly selected genes from the dataset; this background was about one enriched GO term for every three genes [34]. When the number of enriched GO terms is more than five fold over background, this is strong evidence for enrichment [34].

### Phosphopeptide Summation into Phosphorylation Sites

For phosphorylation site analyses, peptide peak intensity values were summed based on sequence homology and phosphorylation site, independent of the presence or absence of oxidized methionine. In cases where conserved sequences did not allow unambiguous assignment to a particular protein, the peptide name either retained multiple names, for example “FYN 420; LCK 394; SRC 419; YES1 426,” or were merged into all possible larger peptides, for example MAPKs and C-terminal inhibitory phosphorylations on SRC, FYN, and YES1 (referred to as inclusively summed).

### Transplantation of Neuroblastoma Cells into Chick Embryos

Four neuroblastoma cell lines (LAN6, SK-N-BE(2), SMS-KCN, and SY5Y) were cultured in 25 μL hanging drops containing approximately 5,000 neuroblastoma cells. Cells were transplanted into the neural crest of developing chick embryos to determine if these cells could survive transplantation and subsequently integrate into the migration pathways of the chick neural crest cells. Of the 14 embryos that were injected, 10 survived the transplantation process: three of these were injected with LAN6 cells, two with SK-N-BE(2) cells, three with SMS-KCN cells, and two with SY5Y cells. Fluorescent imaging of embryo sections showed that all four cell lines were successfully transplanted and could be located within various areas of the embryo with the use of GFP infection with adeno-associated virus that expresses GFP (AAV-GFP, a gift from Dr. D. Poulsen, University of Montana), anti-GFP (Rockland, Gilbertsville, PA), anti-ERGIC (Alexis Biochemicals, U.S.), and fluorescent secondary antibodies (Alexa Fluor 514 goat anti-mouse and Alexa Fluor 488 chicken anti-rabbit from Invitrogen Molecular Probes, U.S.). Further details of embro transplantation methods are provided in S1 Text.

## Supporting Information

S1 Movie Movie of t-SNE SED embedding plotted in three dimensions. PPI edges were filtered to show interactions only among proteins that cluster together. The movie was made from Cytoscape using RCytoscape to iteratively rotate and zoom the network nodes, plotted using coordinates derived from t-SNE SED embedding. Nodes and edges are graphed as in Figs 1 and S1.
(M4V)

S1 Fig Neuroblastoma protein-protein interaction (PPI) network. The size and color of nodes is scaled to graph total phosphopeptides detected for each protein; blue represents phosphorylation on inhibitory sites, yellow, all other sites. Edge thickness represents a quantity (weight) that indicates the strength of evidence for interactions. There were 192 isolated nodes without edges in the network.
(PDF)

S2 Fig Neuroblastoma network properties. (**A**) The neuroblastoma network obeyed the power law degree distribution typical of scale-free biological networks: α = 1.170; R^2^ = 0.795 for all degrees, α = 1.496, R^2^ = 0.820 for degrees > 10. The entire neuroblastoma phosphoproteomic network of 1622 proteins and 18728 interactions has a clustering coefficient of 0.167 and obeys the power law degree distribution typical of scale-free biological networks. This clustering coefficient, the network diameter of 7 (the longest length between connected nodes), and mean path length of 2.78, is consistent with the small-world effect, which is a property of real biological networks. Thus, the highly interconnected network of phosphorylated proteins in neuroblastoma indicates a robust biological network as opposed to a sparse or random selection of proteins [128]. (**B**) The most highly interconnected region of the neuroblastoma phosphoproteomic PPI network (identified by the Cytoscape plugin, MCODE) is an almost perfect clique (a group where every node is connected to every other node). The group is made up of the SFKs (LYN, FYN, and SRC), RTKs, EGFR, PDGFRB, KIT, other tyrosine kinases (PTK2, SYK, STAT5A, JAK1, JAK2, ABL1), a tyrosine phosphatase (SHP-2/PTPN11), and other tyrosine kinase signaling effector proteins that contain SH2 and/or SH3 domains. These 27 nodes are in turn connected to 711 nodes, or 44% of the total proteins in the neuroblastoma network shown in S1 Fig. This interconnected group, which is based only on known interactions (from PPI databases) among all proteins detected in our data, is consistent with the hypothesis that tyrosine kinases, tyrosine phosphatases, and SH2-domain-containing proteins, which expanded during evolution when animals became multicellular [19] (Liu and Nash, 2012), are positioned to control the network of phosphorylated proteins identified in neuroblastoma cell lines.
(PDF)

S3 Fig Heat map showing the relative total phosphopeptide amounts for all RTKs detected in neuroblastoma samples on a blue-yellow scale (black represents NA; key, left). Rows were sorted by hierarchical clustering using a modified distance function that can handle missing values.
(PDF)

S4 Fig Neuroblastoma cells migrate along stereotypic neural crest migration pathways to colonize most trunk neural crest derivatives and differentiate into peripheral neurons. (**A, top**) GFP-expressing neuroblastoma cells, transplanted into chick embryos, express the neural crest marker HNK, and colonize derivatives ventral to the dorsal aorta as well as progenitor zones within the dorsal root ganglia (DRG) including the dorsal pole and lateral perimeter [129]. (**A, bottom**) Neuroblastoma cells give rise to afferents in the dorsal root and sympathetic ganglia that exhibit normal neuronal morphology (including dorsal and ventral extensions) and colocalize with the neuronal marker Tuj-1. (**B**) Number of neuroblastoma cells according to their final migration location within the chick embryo and cell type. 164 LAN-6; 102 SK-N-BE(2); 86 SMS-KCN; and 142 SY5Y cells were detected in chick embryos after transplantation using human-specific anti-ER-Golgi intermediate compartment marker (ERGIC-53; see Materials and Methods). All cell lines migrated to most trunk neural crest derivatives within the developing chick embryo. The number of cells detected in each embryonic location is shown. Cells whose location could not be unambiguously determined were classified as unknown/random. There were differences in migration patterns for different cell lines, but experiment-to-experiment variation in migration patterns was high, so differences did not attain statistical significance.
(PDF)

S5 Fig Evaluation of clusters. Clusters identified from Spearman, Euclidean, or SED t-SNE embeddings were validated by internal and external evaluations as described [34]. Compared to random clusters, clusters identified from Spearman, Euclidean, or SED t-SNE embeddings (indicated by labels on box plots), had lower percent NA (**A**), higher index (**B**), more edges per cluster (**C**), more edge weight per cluster (**D**), more GO term mean count over expected (**E**), and more GO terms per gene (**F**) than the random clusters. All graphs except A are plotted on a log scale. Statistical significance determined by the Welch two-sided t-test comparing random clusters to all t-SNE clusters is p < 0.0001 (A); p < 0.000001 (B); p < 0.00005 (C); p < 0.0002 (D); p < 0.00005 (E); and p < 0.006 (F).
(PDF)

S6 Fig Additional “hard” filtered clusters containing RTKs. Proteins that cluster in all three dissimilarity representations (Spearman, Euclidean, and SED) with EPHA2 (**A**), PDGFRB (**B**), and KIT (**C**), graphed as PPI networks (left) and heat maps (right) as in Fig 1. Similar statistical relationships predict previously uncharacterized interactions between EPHA2 and the SH2-containing RAS-GEF SH2D3C (**A**), PDGFRB and the Rho-GEF, intersectin 2 (ITSN2) and the adaptor, STAM2 (**B**), and the RTKs, KIT and ROR1 (**C**).
(PDF)

S7 Fig Co-cluster correlation edge network. This graph is similar to Fig 2 except that edges represent Spearman correlation ≧ absolute value of 0.5; positive correlations are yellow; negative, blue. Nodes are graphed as in Figs 1 and S1. Proteins that have no correlation edges within clusters to which they belong are shown on the bottom right.
(PDF)

S8 Fig Heat maps showing relative phosphorylation among members of collaborative groups shown in Fig 4. ALK group (**A**) and EGFR group (**B**), graphed as heat maps as in Fig 1 except proteins were sorted by hierarchical clustering using a modified distance function as in S3 Fig.
(PDF)

S9 Fig Fold change in response to RTK stimulation or inhibition. Shown are changes of more than twofold from representative experiments where peak intensity was measured for treatment and control conditions in the same experiment with cell lines and treatments indicated on column labels as in Fig 4. Individual phosphorylation site changes are shown for RTKs (**A**), and other tyrosine kinases (**B**). Fold changes are graphed on a blue-yellow color heat map as in Fig 4.
(PDF)

S10 Fig Cell fractionation strategy. Fractionation strategy for cell fractions as performed previously [32; 33]. Endosomes and other organelles were fractionated by sedimentation velocity (proportional to mass) followed by equilibrium density by floatation. The mass vs. density graph in the center summarizes the localization of lysosomes and three signaling receptors in endosomes (p75NTR, pTrkA/NTRK1, PAC1) [32]. These data were used to define fractions shown on the graph at right (lys, lysosomes; E1 and E2, endosomes). Inset shows a western blot using anti-phospho-ALK in endosome fractions after 10 min pleiotropin (PTN) treatment of SMS-KCN cells.
(PDF)

S11 Fig Co-cluster correlation network (CCCN) for all tyrosine phosphorylation sites detected in neuroblastoma samples. All tyrosine phosphorylation sites detected in two or more samples. Node size and color indicate amount of phosphorylation on each site in all samples, with inhibitory sites blue, all others yellow, as in Fig 1. Edges represent Spearman correlation ≧ absolute value of 0.5, with positive correlation represented as yellow, negative correlation, blue, filtered to show only co-clustered phosphorylation sites. Negative correlation edges were transformed by the formula, *edge weight = e* ^(20 * correlation)^ to cause the edge-weighted, spring-embedded layout to graph negatively correlated nodes far apart. Peptides were inclusively summed for this phosphorylation site network (see Materials and Methods), rather than exclusively summed for the total phosphorylation protein network (S1 Fig). This inclusive method assigns phosphorylation sites that have conserved phosphopeptides with identical sequence to all potential phosphorylation sites. Phosphorylation sites without edges are not shown.
(PDF)

S12 Fig RTK phosphorylation site enrichment in endosomes for LAN-6 cells (A) and NTRK1-expressing SK-N-BE(2) cells (B). Enrichment of phosphorylation sites in endosome and DRM fractions was calculated as the ratio of amounts in endosomes or DRMs vs. the average in all other fractions and samples from that cell line, graphed as heat maps as in Fig 5G.
(PDF)

S1 Text Detailed methods for transplation of neuroblastoma cells into chick embryos. (PDF)

S1 Dataset Gene ontology summaries of clusters. (ZIP)

S2 Dataset CFN and CCCNs for proteins and phosphorylation sites. (ZIP)

S3 Dataset Total protein and phosphorylation site data. (ZIP)
