# Supplementary figures and images for: Neuroblastoma Tyrosine Kinase Signaling Networks Involve FYN and LYN in Endosomes and Lipid Rafts
Source: PLoS Comput Biol. 2015 Apr 17;11(4):e1004130. doi: 10.1371/journal.pcbi.1004130 (PMC4401789; doi:10.1371/journal.pcbi.1004130)

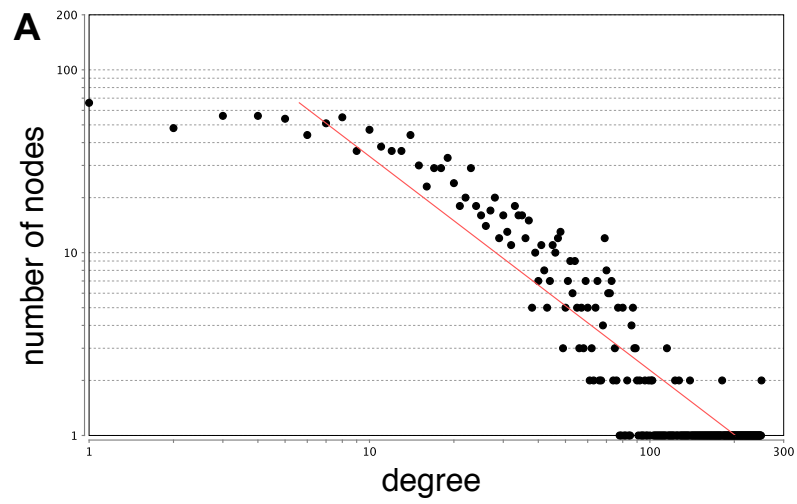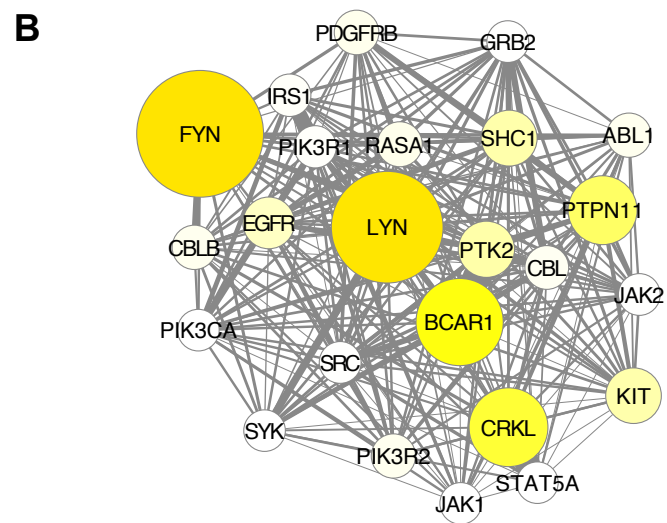

Figure S2

Supplement: S2 Fig — (A) The neuroblastoma network obeyed the power law degree distribution typical of scale-free biological networks: α = 1.170; R2 = 0.795 for all degrees, α = 1.496, R2 = 0.820 for degrees > 10. The entire neuroblastoma phosphoproteomic network of 1622 proteins and 18728 interactions has a clustering coefficient of 0.167 and obeys the power law degree distribution typical of scale-free biological networks. This clustering coefficient, the network diameter of 7 (the longest length between connected nodes), and mean path length of 2.78, is consistent with the small-world effect, which is a property of real biological networks. Thus, the highly interconnected network of phosphorylated proteins in neuroblastoma indicates a robust biological network as opposed to a sparse or random selection of proteins [128]. (B) The most highly interconnected region of the neuroblastoma phosphoproteomic PPI network (identified by the Cytoscape plugin, MCODE) is an almost perfect clique (a group where every node is connected to every other node). The group is made up of the SFKs (LYN, FYN, and SRC), RTKs, EGFR, PDGFRB, KIT, other tyrosine kinases (PTK2, SYK, STAT5A, JAK1, JAK2, ABL1), a tyrosine phosphatase (SHP-2/PTPN11), and other tyrosine kinase signaling effector proteins that contain SH2 and/or SH3 domains. These 27 nodes are in turn connected to 711 nodes, or 44% of the total proteins in the neuroblastoma network shown in S1 Fig. This interconnected group, which is based only on known interactions (from PPI databases) among all proteins detected in our data, is consistent with the hypothesis that tyrosine kinases, tyrosine phosphatases, and SH2-domain-containing proteins, which expanded during evolution when animals became multicellular [19] (Liu and Nash, 2012), are positioned to control the network of phosphorylated proteins identified in neuroblastoma cell lines. (PDF) [file pcbi.1004130.s003.pdf]

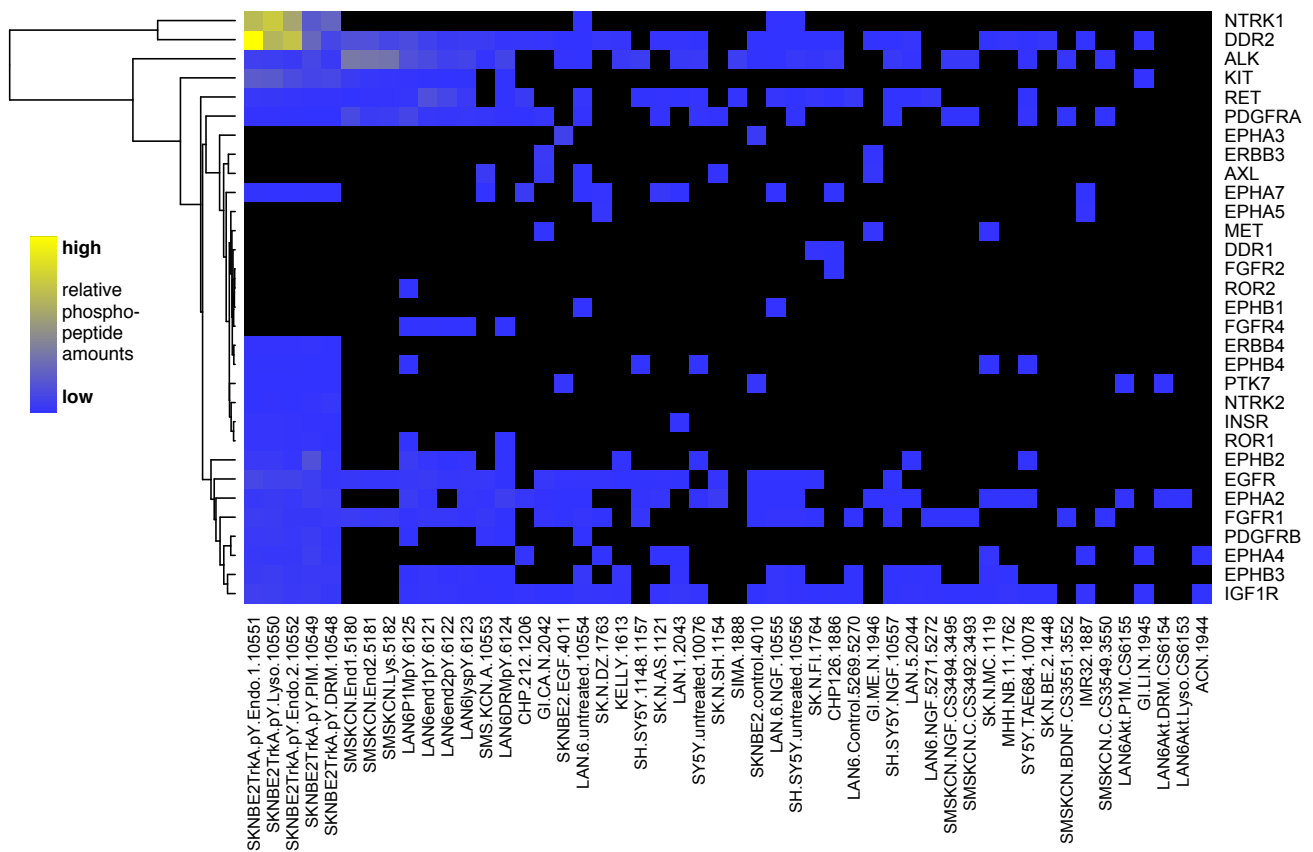

Figure S3

Supplement: S3 Fig — Rows were sorted by hierarchical clustering using a modified distance function that can handle missing values. (PDF) [file pcbi.1004130.s004.pdf]

**A**

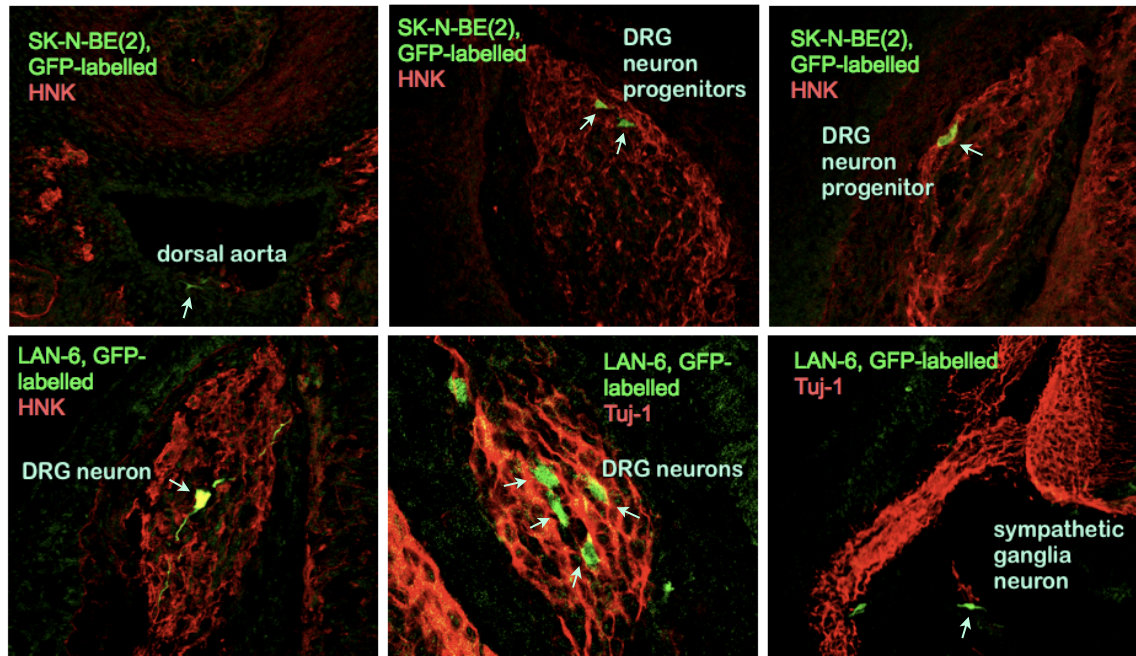

**B**

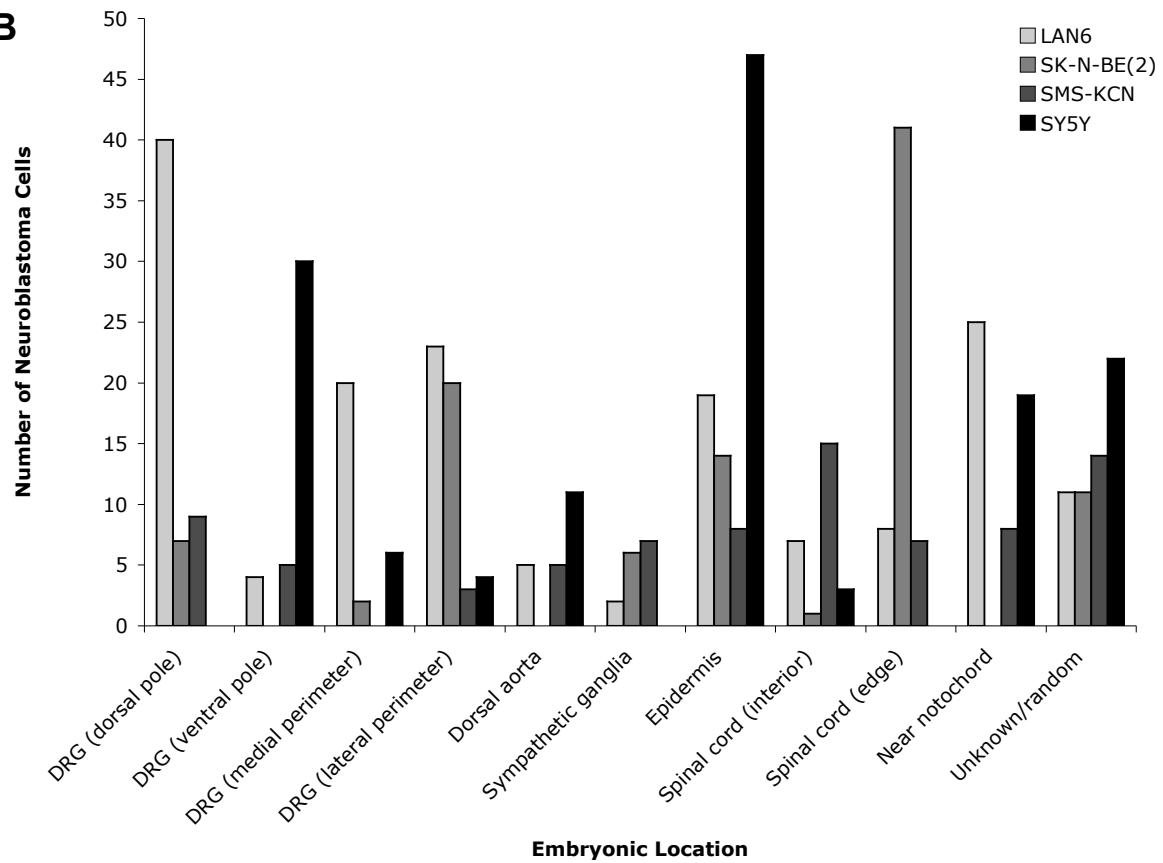

Figure S4

Supplement: S4 Fig — (A, top) GFP-expressing neuroblastoma cells, transplanted into chick embryos, express the neural crest marker HNK, and colonize derivatives ventral to the dorsal aorta as well as progenitor zones within the dorsal root ganglia (DRG) including the dorsal pole and lateral perimeter [129]. (A, bottom) Neuroblastoma cells give rise to afferents in the dorsal root and sympathetic ganglia that exhibit normal neuronal morphology (including dorsal and ventral extensions) and colocalize with the neuronal marker Tuj-1. (B) Number of neuroblastoma cells according to their final migration location within the chick embryo and cell type. 164 LAN-6; 102 SK-N-BE(2); 86 SMS-KCN; and 142 SY5Y cells were detected in chick embryos after transplantation using human-specific anti-ER-Golgi intermediate compartment marker (ERGIC-53; see Materials and Methods). All cell lines migrated to most trunk neural crest derivatives within the developing chick embryo. The number of cells detected in each embryonic location is shown. Cells whose location could not be unambiguously determined were classified as unknown/random. There were differences in migration patterns for different cell lines, but experiment-to-experiment variation in migration patterns was high, so differences did not attain statistical significance. (PDF) [file pcbi.1004130.s005.pdf]

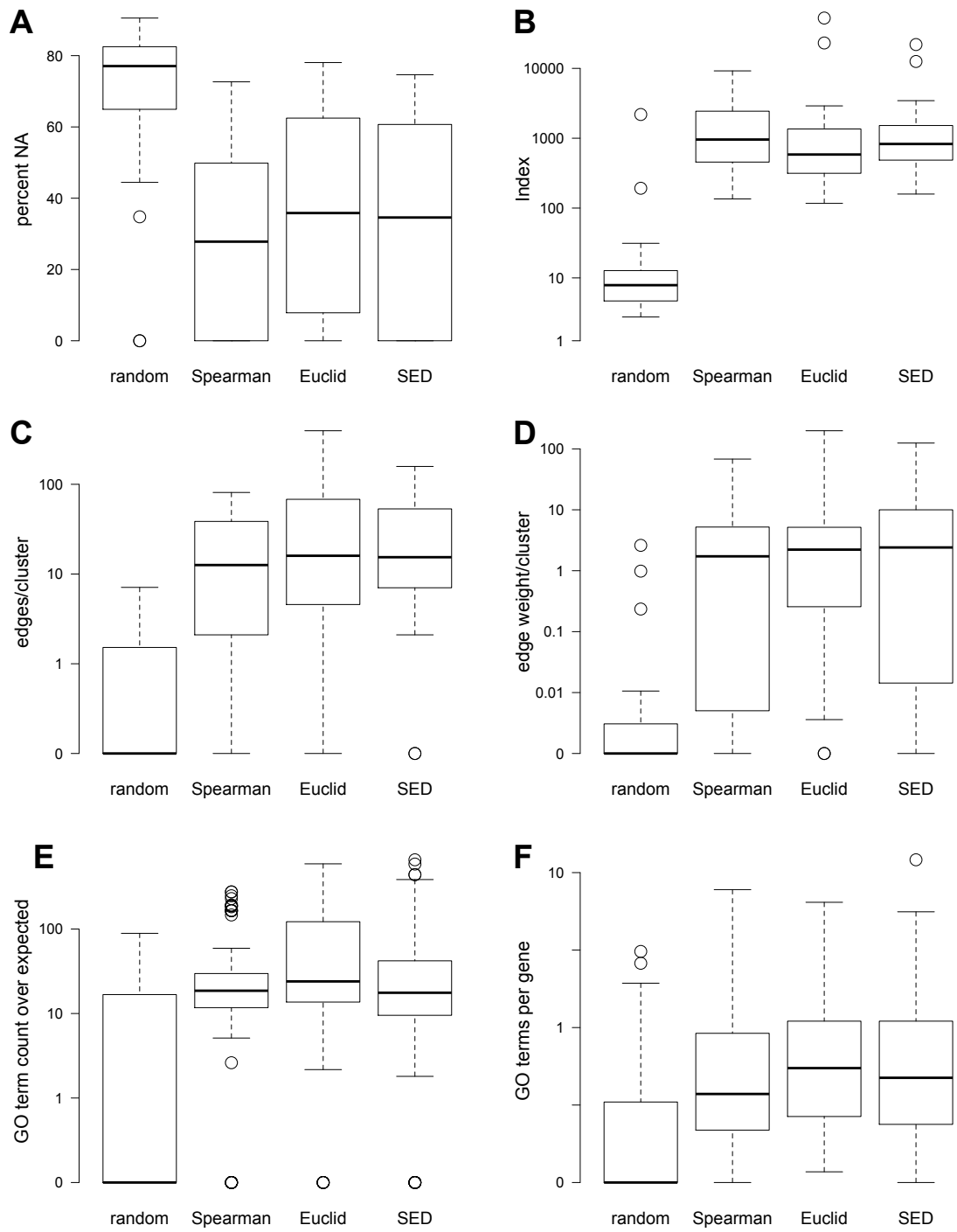

Figure S5

Supplement: S5 Fig — Clusters identified from Spearman, Euclidean, or SED t-SNE embeddings were validated by internal and external evaluations as described [34]. Compared to random clusters, clusters identified from Spearman, Euclidean, or SED t-SNE embeddings (indicated by labels on box plots), had lower percent NA (A), higher index (B), more edges per cluster (C), more edge weight per cluster (D), more GO term mean count over expected (E), and more GO terms per gene (F) than the random clusters. All graphs except A are plotted on a log scale. Statistical significance determined by the Welch two-sided t-test comparing random clusters to all t-SNE clusters is p < 0.0001 (A); p < 0.000001 (B); p < 0.00005 (C); p < 0.0002 (D); p < 0.00005 (E); and p < 0.006 (F). (PDF) [file pcbi.1004130.s006.pdf]

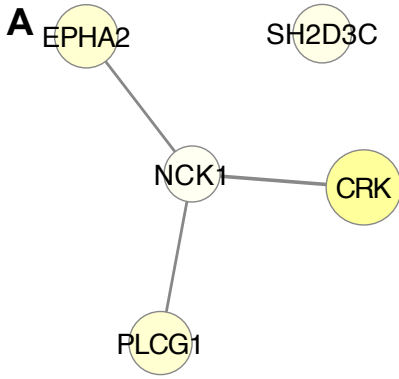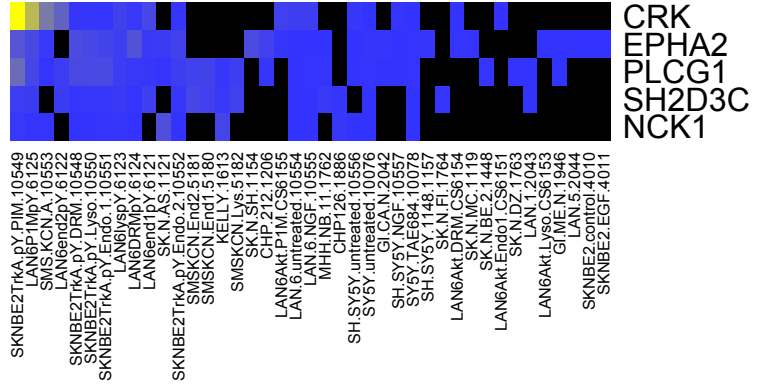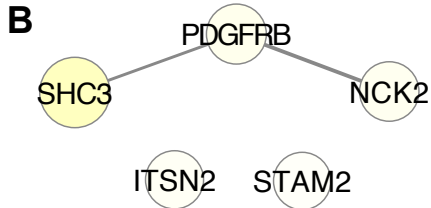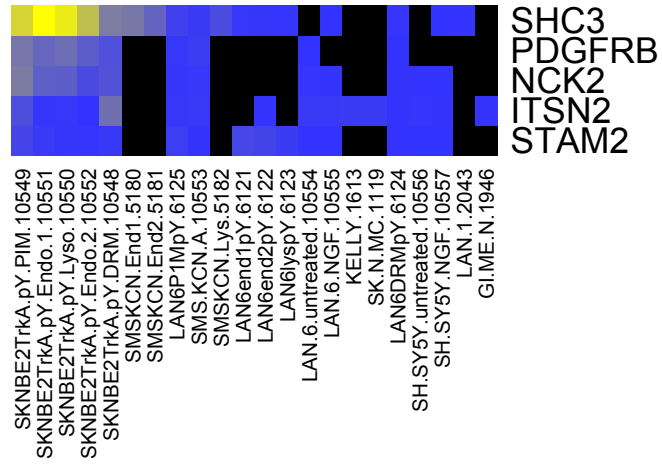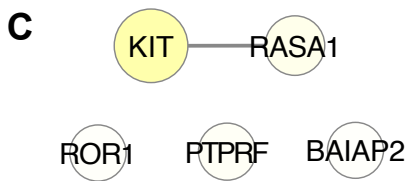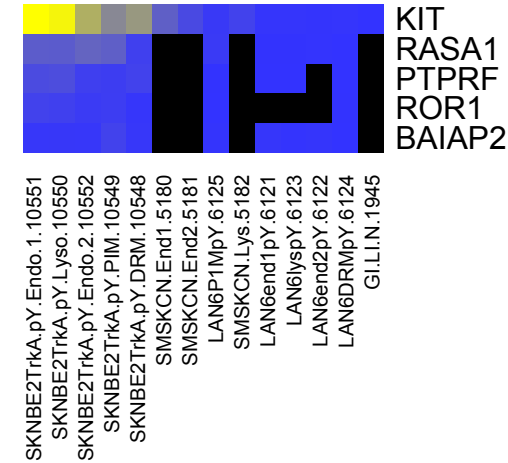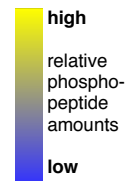

Figure S6

Supplement: S6 Fig — Proteins that cluster in all three dissimilarity representations (Spearman, Euclidean, and SED) with EPHA2 (A), PDGFRB (B), and KIT (C), graphed as PPI networks (left) and heat maps (right) as in Fig 1. Similar statistical relationships predict previously uncharacterized interactions between EPHA2 and the SH2-containing RAS-GEF SH2D3C (A), PDGFRB and the Rho-GEF, intersectin 2 (ITSN2) and the adaptor, STAM2 (B), and the RTKs, KIT and ROR1 (C). (PDF) [file pcbi.1004130.s007.pdf]

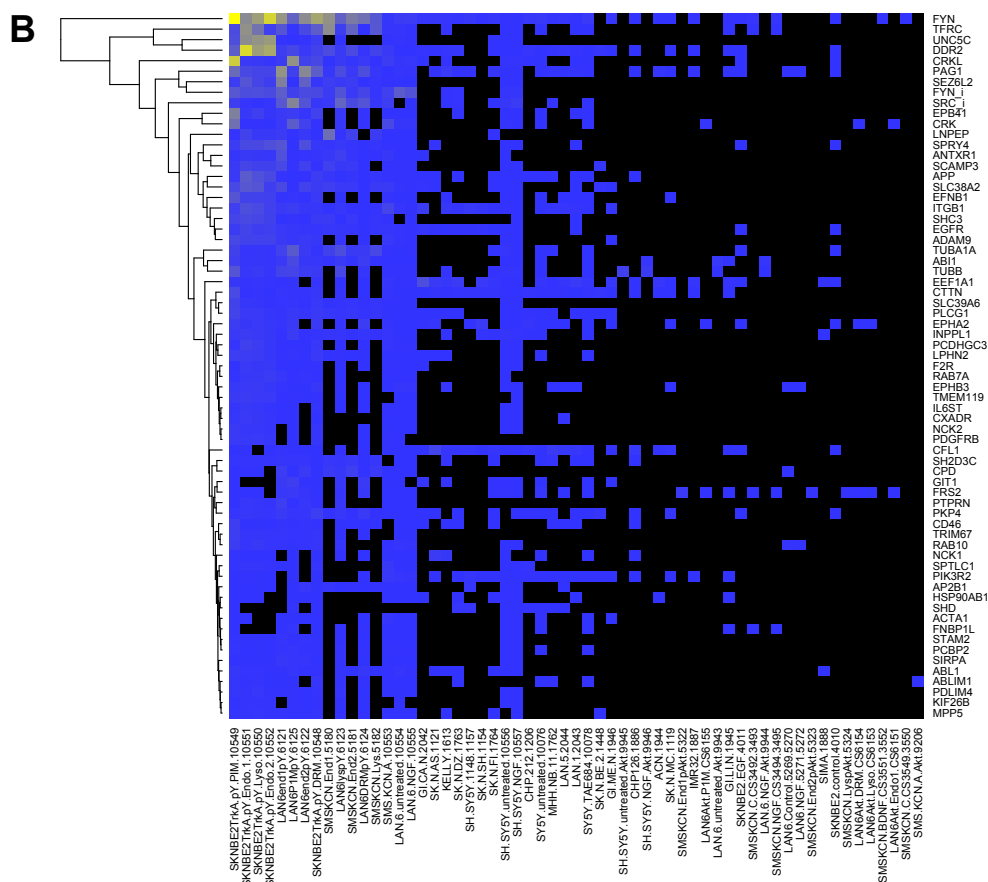

•

Supplement: S8 Fig — ALK group (A) and EGFR group (B), graphed as heat maps as in Fig 1 except proteins were sorted by hierarchical clustering using a modified distance function as in S3 Fig. (PDF) [file pcbi.1004130.s009.pdf]

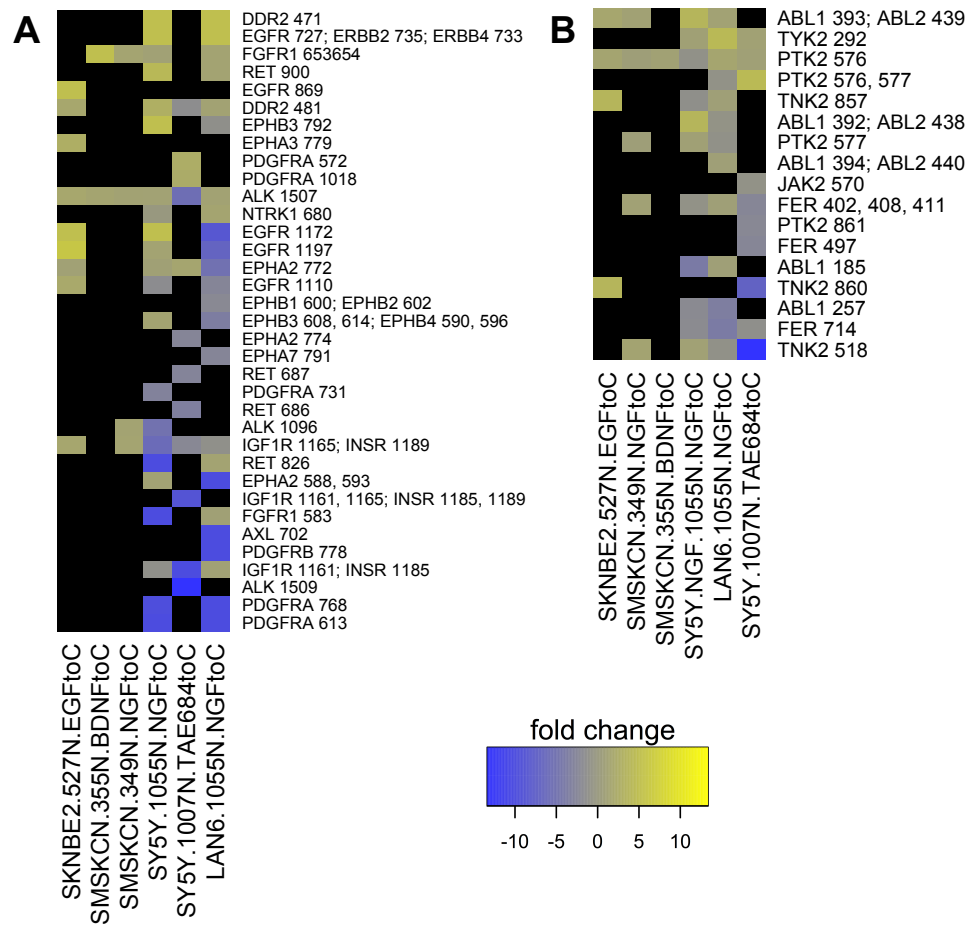

Figure S9

Supplement: S9 Fig — Shown are changes of more than twofold from representative experiments where peak intensity was measured for treatment and control conditions in the same experiment with cell lines and treatments indicated on column labels as in Fig 4. Individual phosphorylation site changes are shown for RTKs (A), and other tyrosine kinases (B). Fold changes are graphed on a blue-yellow color heat map as in Fig 4. (PDF) [file pcbi.1004130.s010.pdf]

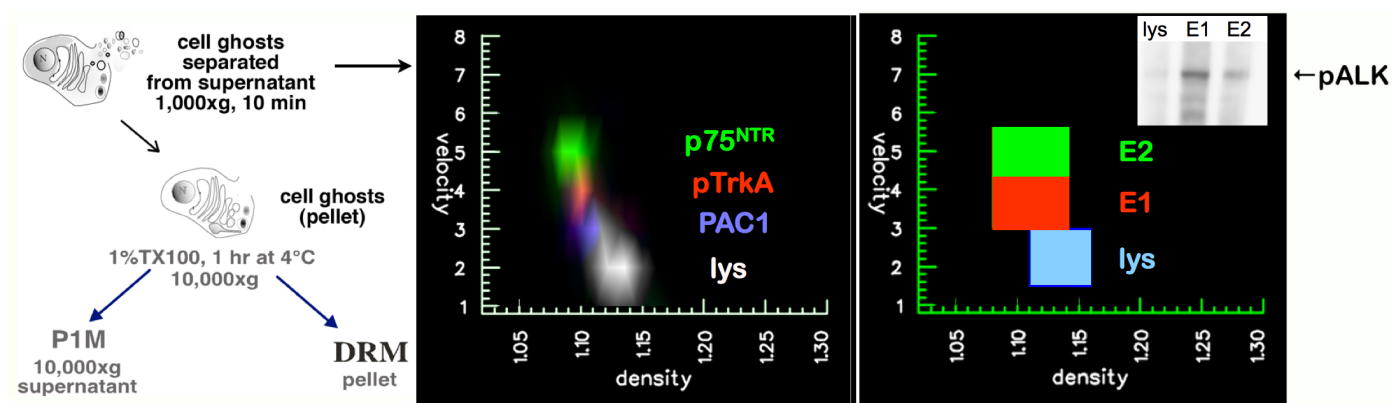

Figure S10

Supplement: S10 Fig — Fractionation strategy for cell fractions as performed previously [32; 33]. Endosomes and other organelles were fractionated by sedimentation velocity (proportional to mass) followed by equilibrium density by floatation. The mass vs. density graph in the center summarizes the localization of lysosomes and three signaling receptors in endosomes (p75NTR, pTrkA/NTRK1, PAC1) [32]. These data were used to define fractions shown on the graph at right (lys, lysosomes; E1 and E2, endosomes). Inset shows a western blot using anti-phospho-ALK in endosome fractions after 10 min pleiotropin (PTN) treatment of SMS-KCN cells. (PDF) [file pcbi.1004130.s011.pdf]

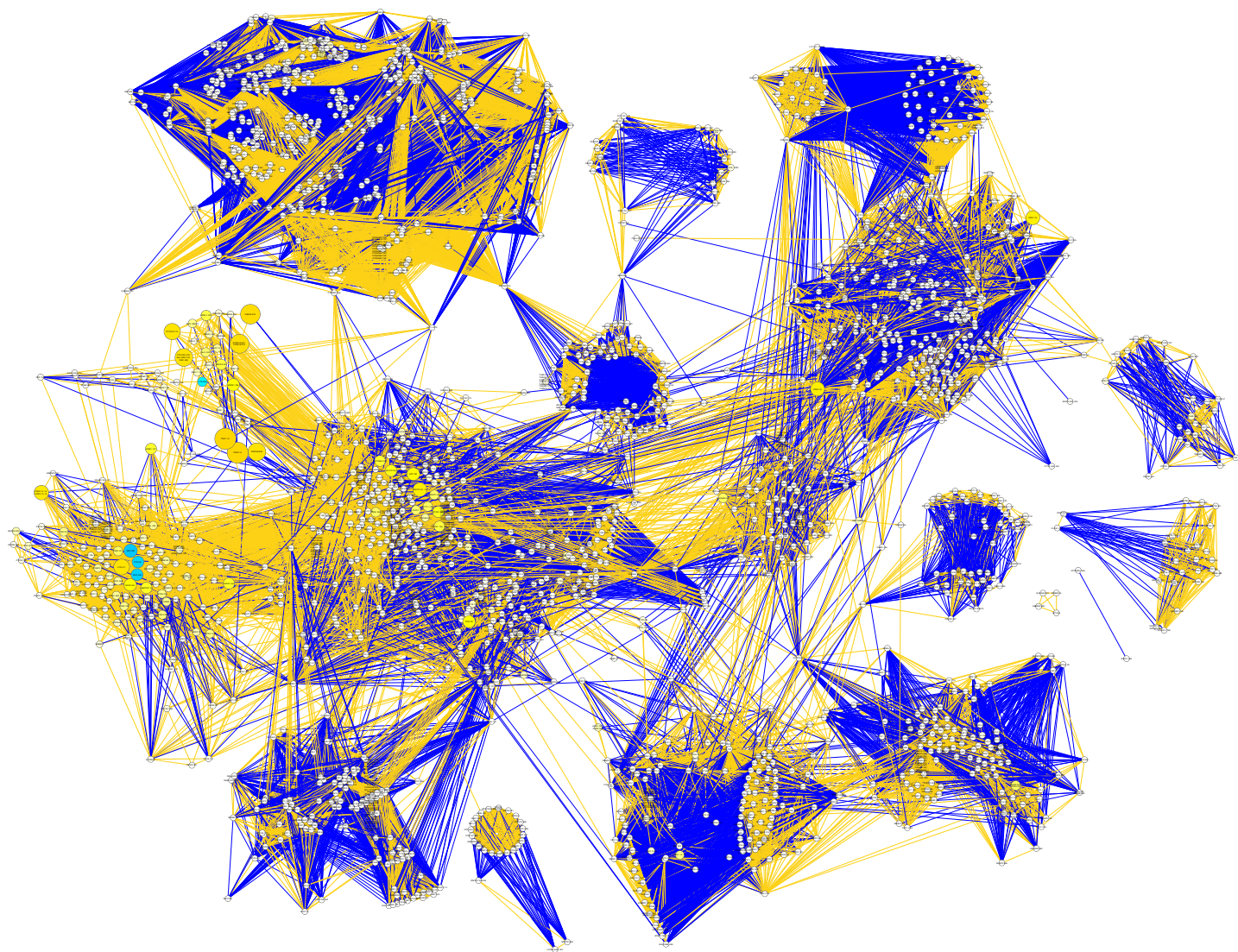

Figure S11

Supplement: S11 Fig — All tyrosine phosphorylation sites detected in two or more samples. Node size and color indicate amount of phosphorylation on each site in all samples, with inhibitory sites blue, all others yellow, as in Fig 1. Edges represent Spearman correlation ≧ absolute value of 0.5, with positive correlation represented as yellow, negative correlation, blue, filtered to show only co-clustered phosphorylation sites. Negative correlation edges were transformed by the formula, edge weight = e (20 * correlation) to cause the edge-weighted, spring-embedded layout to graph negatively correlated nodes far apart. Peptides were inclusively summed for this phosphorylation site network (see Materials and Methods), rather than exclusively summed for the total phosphorylation protein network (S1 Fig). This inclusive method assigns phosphorylation sites that have conserved phosphopeptides with identical sequence to all potential phosphorylation sites. Phosphorylation sites without edges are not shown. (PDF) [file pcbi.1004130.s012.pdf]

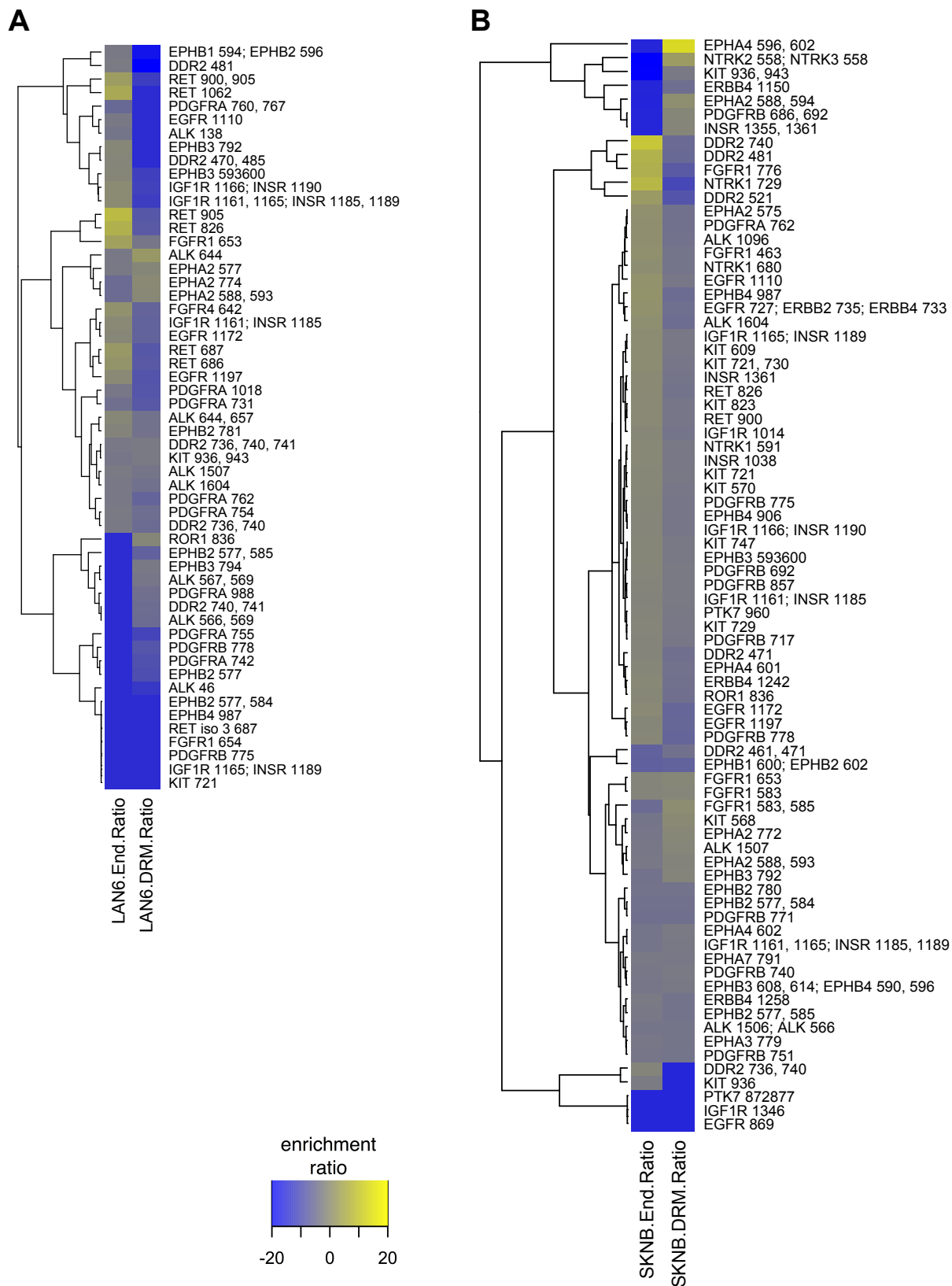

Figure S12

Supplement: S12 Fig — Enrichment of phosphorylation sites in endosome and DRM fractions was calculated as the ratio of amounts in endosomes or DRMs vs. the average in all other fractions and samples from that cell line, graphed as heat maps as in Fig 5G. (PDF) [file pcbi.1004130.s013.pdf]
