# Supplementary material for: Neuroblastoma Tyrosine Kinase Signaling Networks Involve FYN and LYN in Endosomes and Lipid Rafts
Source: PLoS Comput Biol. 2015 Apr 17;11(4):e1004130. doi: 10.1371/journal.pcbi.1004130 (PMC4401789; doi:10.1371/journal.pcbi.1004130)
Supplement: S1 Text — (PDF) [file pcbi.1004130.s014.pdf]

Supplemental Materials and Methods

***In ovo* Microinjection of Neuroblastoma Cells**

Fertilized specific pathogen-free (SPF) chicken eggs were obtained from Charles River Laboratories, MA and were incubated for approximately 50-55 hours in a rocking incubator at 37°C with a water pan to maintain humidity until embryos reached Hamburger/Hamilton stage 15. A wide-bore needle and syringe were used to remove 4-5 mL of albumin from the narrow end of the egg. Using a pair of curved scissors, a window was cut in the eggshell above the embryo. A capillary glass needle (Drummond Scientific Company, U.S., 3-000-210-G8) was pulled on a single stage glass microelectrode puller (Narishige Model PP-830, Tritech Research, U.S.). This needle was loaded with approximately 1 µL of 5% fast green FCF (Sigma, U.S.) and 9 µL from the 25 µL neuroblastoma hanging drop, containing approximately 5,000 cells, using a microinjector (Drummond Scientific Company, U.S., 3-000-510-X) and a micromanipulator system (SISKIYOU Corporation, Grants Pass, OR). Using the micromanipulator adjustments, the loaded needle was aligned with and inserted into the neural tube of the developing embryo. On occasion, approximately 0.5-1 mL of 5% fast green was injected into the yolk beneath the embryo for visualization purposes. The mixture of neuroblastoma cells and 5% fast green was then injected into the embryo until the entire neural tube was filled (approximately 2-3 µL per injection); one loaded needle was typically used for 3-4 embryo injections. Following the cell transplantation process, the eggshells were sealed with cellophane tape and returned to the incubator for an additional two to four days.

Harvesting and Sectioning Embryos

Embryos were harvested after 2-4 days of incubation, at Hamburger/Hamilton stage 21-27, noting the stage of development for each embryo. Embryos were dissected from the vitelline and amnion, decapitated and eviscerated, then transferred to scintillation vials containing 2.5 mL of 4% paraformaldehyde fixative and rotated at 4 °C for one to four hours, depending on embryonic stage of development (minimum fixation time in hours = 0.5 × the total number of days incubated; maximum fixation time in hours = 1 × the total number of days incubated). The fixative was then aspirated from the vials and embryos were washed twice with cold 1X PBS, 10 minutes per wash. Embryos were then dehydrated in increasing concentrations of sucrose in 1X PBS (5%, 10%, and 30% sucrose in 1X PBS); embryos rotated in these solutions at 4 °C until they sank in the solution, at which point the embryos were transferred to a vial containing the subsequent solution. Following this dehydration process, embryos were transferred to vials containing a 1:1 solution of 30% sucrose in 1X PBS and Tissue-Tek Optimal Cutting Temperature (O.C.T.) compound (VWR International, U.S.), and rotated in this solution at 4 °C for two hours. Embryos were then transferred to vials containing pure O.C.T. and rotated at 4 °C for two hours. Finally, embryos were embedded in O.C.T. in Peel-A-Way™ disposable square (22mm x 22mm) embedding molds (Dumond Chemicals, U.S.), frozen in a dry ice and ethanol bath, and were stored at -80 °C.

Embedded embryos were sectioned using a 0620-SME Shandon Cryostat (Life Sciences International, England). Tissue sections were cut 16 µm thick at a working temperature of -20 °C. Sections were placed on Fisherbrand Superfrost/Plus microscope slides (Fisher Scientific, U.S.), typically with 20-25 tissue sections per slide. Slides of tissue sections were stored at -80 °C until utilized in the immunohistochemistry procedure.

Immunohistochemistry

Microscope slides of fixed, sectioned embryos were incubated in 1X TBS for 20 minutes followed by a 45 minute incubation in permeabilization solution (0.1% saponin, 1% BSA, 2% goat serum in 1X PBS), both conducted at room temperature. Slides were then incubated in respective primary antibodies, diluted in permeabilization solution, for three hours at room temperature. Following this incubation period, slides were washed 4 times with permeabilization solution, then incubated in corresponding secondary fluorescent antibodies then washed four times. Coverslips were mounted to the microscope slides using Vectashield mounting medium (Vector Laboratories, U.S.). Immunofluorescence was examined using the 40x oil-immersion objective on a Nikon E800 microscope and Openlab 5 Software (Improvision, U.S.). Neuronal markers Tuj1 (antibody from R & D Systems) and BEN (antibody from Developmental Hybridoma Bank, University of Iowa) were used to identify the major structures within the chick embryo.
