# Supplementary material for: Neuroblastoma Tyrosine Kinase Signaling Networks Involve FYN and LYN in Endosomes and Lipid Rafts
Source: PLoS Comput Biol. 2015 Apr 17;11(4):e1004130. doi: 10.1371/journal.pcbi.1004130 (PMC4401789; doi:10.1371/journal.pcbi.1004130)
Supplement: S1 Fig — The size and color of nodes is scaled to graph total phosphopeptides detected for each protein; blue represents phosphorylation on inhibitory sites, yellow, all other sites. Edge thickness represents a quantity (weight) that indicates the strength of evidence for interactions. There were 192 isolated nodes without edges in the network. (PDF) [file pcbi.1004130.s002.pdf]

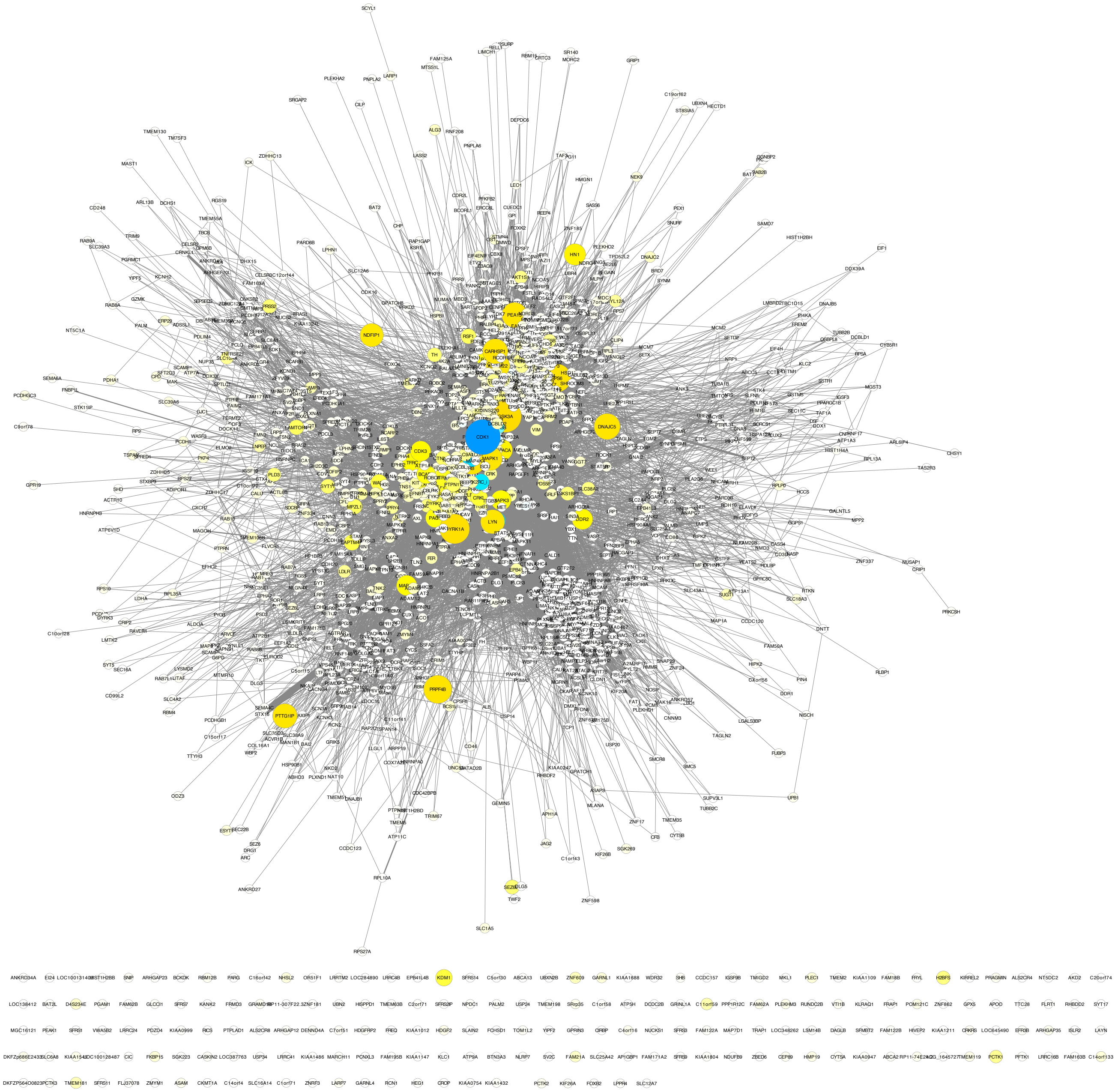

Figure S1
