# Supplementary material for: Neuroblastoma Tyrosine Kinase Signaling Networks Involve FYN and LYN in Endosomes and Lipid Rafts
Source: PLoS Comput Biol. 2015 Apr 17;11(4):e1004130. doi: 10.1371/journal.pcbi.1004130 (PMC4401789; doi:10.1371/journal.pcbi.1004130)
Supplement: S7 Fig — This graph is similar to Fig 2 except that edges represent Spearman correlation ≧ absolute value of 0.5; positive correlations are yellow; negative, blue. Nodes are graphed as in Figs 1 and S1. Proteins that have no correlation edges within clusters to which they belong are shown on the bottom right. (PDF) [file pcbi.1004130.s008.pdf]

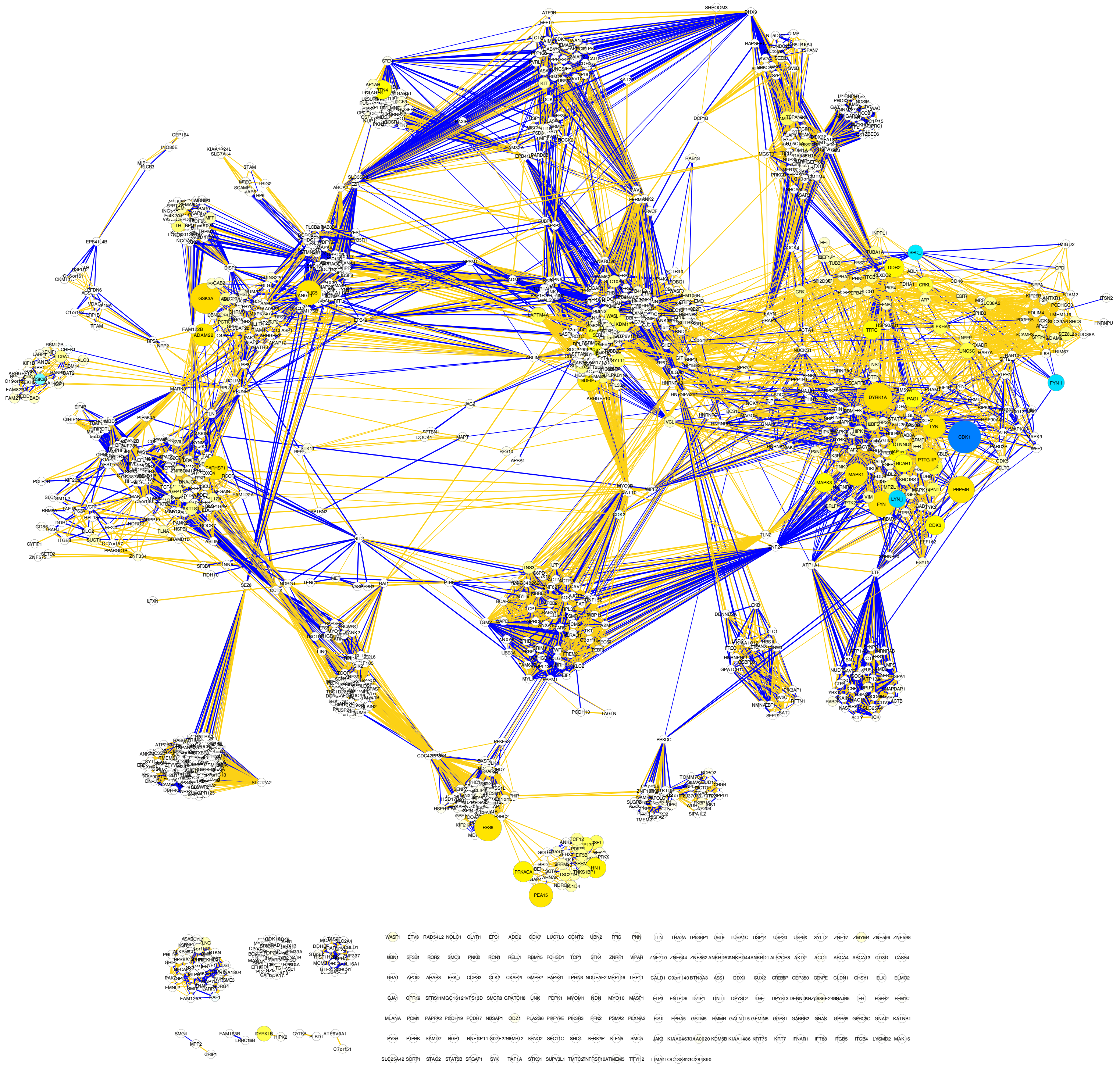

Figure S7
